# Integrated bioinformatics and network pharmacology to identify the therapeutic target and molecular mechanisms of Huangqin decoction on ulcerative Colitis

**DOI:** 10.1038/s41598-021-03980-8

**Published:** 2022-01-07

**Authors:** Yi Wu, Xinqiao Liu, Guiwei Li

**Affiliations:** 1 First Teaching Hospital of Tianjin University of Traditional Chinese Medicine, Tianjin, 300000 China; 2 National Clinical Research Center for Chinese Medicine Acupuncture and Moxibustion, Tianjin, 300000 China

**Keywords:** Computational biology and bioinformatics, Molecular biology, Gastroenterology

## Abstract

Huangqin decoction (HQD) is a Traditional Chinese Medicine formula for ulcerative colitis. However, the pharmacology and molecular mechanism of HQD on ulcerative colitis is still unclear. Combined microarray analysis, network pharmacology, and molecular docking for revealing the therapeutic targets and molecular mechanism of HQD against ulcerative colitis. TCMSP, DrugBank, Swiss Target Prediction were utilized to search the active components and effective targets of HQD. Ulcerative colitis effective targets were obtained by microarray data from the GEO database (GSE107499). Co-targets between HQD and ulcerative colitis are obtained by Draw Venn Diagram. PPI (Protein–protein interaction) network was constructed by the STRING database. To obtain the core target, topological analysis is exploited by Cytoscape 3.7.2. GO and KEGG enrichment pathway analysis was performed to Metascape platform, and molecular docking through Autodock Vina 1.1.2 finished. 161 active components with 486 effective targets of HQD were screened. 1542 ulcerative colitis effective targets were obtained with |Log_2_FC|> 1 and adjusted *P*-value < 0.05. The Venn analysis was contained 79 co-targets. Enrichment analysis showed that HQD played a role in TNF signaling pathway, IL-17 signaling pathway, Th17 cell differentiation, etc. IL6, TNF, IL1B, PTGS2, ESR1, and PPARG with the highest degree from PPI network were successfully docked with 19 core components of HQD, respectively. According to ZINC15 database, quercetin (ZINC4175638), baicalein (ZINC3871633), and wogonin (ZINC899093) recognized as key compounds of HQD on ulcerative colitis. PTGS2, ESR1, and PPARG are potential therapeutic targets of HQD. HQD can act on multiple targets through multi-pathway, to carry out its therapeutic role in ulcerative colitis.

## Introduction

Ulcerative Colitis (UC) is one subtype of inflammatory bowel disease (IBD) of unknown aetiology that affects the colorectum, often leads to bloody diarrhoea and abdominal pain^1^. Multiple pathogenesis, such as environmental factors, genetically susceptibility and gut microbiome dysfunction, have been implicated^2^. The mucosal inflammation initiating in the rectum and extending proximally in the colon, and limited to the mucosal layer, contrast to Crohn’s disease (CD) is involving all layers of the bowel wall^1^. Owing to intestinal homeostasis dysfunction and intestinal barrier disruption, may result in long-term relapsing and remitting mucosal inflammation^3^. Patients with ulcerative colitis experience unpredictable flares-up may present fever, tenesmus, anemia, and possibly intestinal perforation, which is an afflicted condition of an individual^4,5^. Despite 5-ASA, rectal corticosteroids, and biological drugs are the main treatment of ulcerative colitis, approximately 15% of patients may require restorative proctocolectomy with ileal pouch-anal anastomosis (IPAA) for medically refractory disease or to treat colonic neoplasia^6,7^. However, with the incidence and prevalence of ulcerative colitis is increasing worldwide, governments should be informed of the management and economic burden of this disease. Therefore, it is urgent to seek more therapeutic armamentariums with fewer adverse side effects for ulcerative colitis.

Traditional Chinese Medicine (TCM) has almost 5000 years of history, which creates many treatments for disease. Huangqin decoction (HQD), a traditional herbal formula from “*Shang Han Lun*” written by Zhang Zhongjing in Han Dynasty, which is used for “heat-dampness” ZHENG (resulting in fever, abdominal pain, and hematochezia) for over 1800 years. HQD consists of four herbs: *Scutellariae Radix* (SR, Huangqin), *Paeoniae Radix Alba* (PRA, Baishao), *Jujubae Fructus* (JF, Dazao), *Glycyrrhiza glabra L.* (GL, Gancao). Recently, some fundamental analysis has proved that HQD may regulate the gut microbiota, suppress Ras-PI3K-Akt-HIF-1α and NF-κB pathways^8^, target ESR1 and EGFR to relieve endothelial dysfunction^9^ to treat ulcerative colitis. However, pharmacology and molecular mechanisms remain unclear.

**Figure 1:**
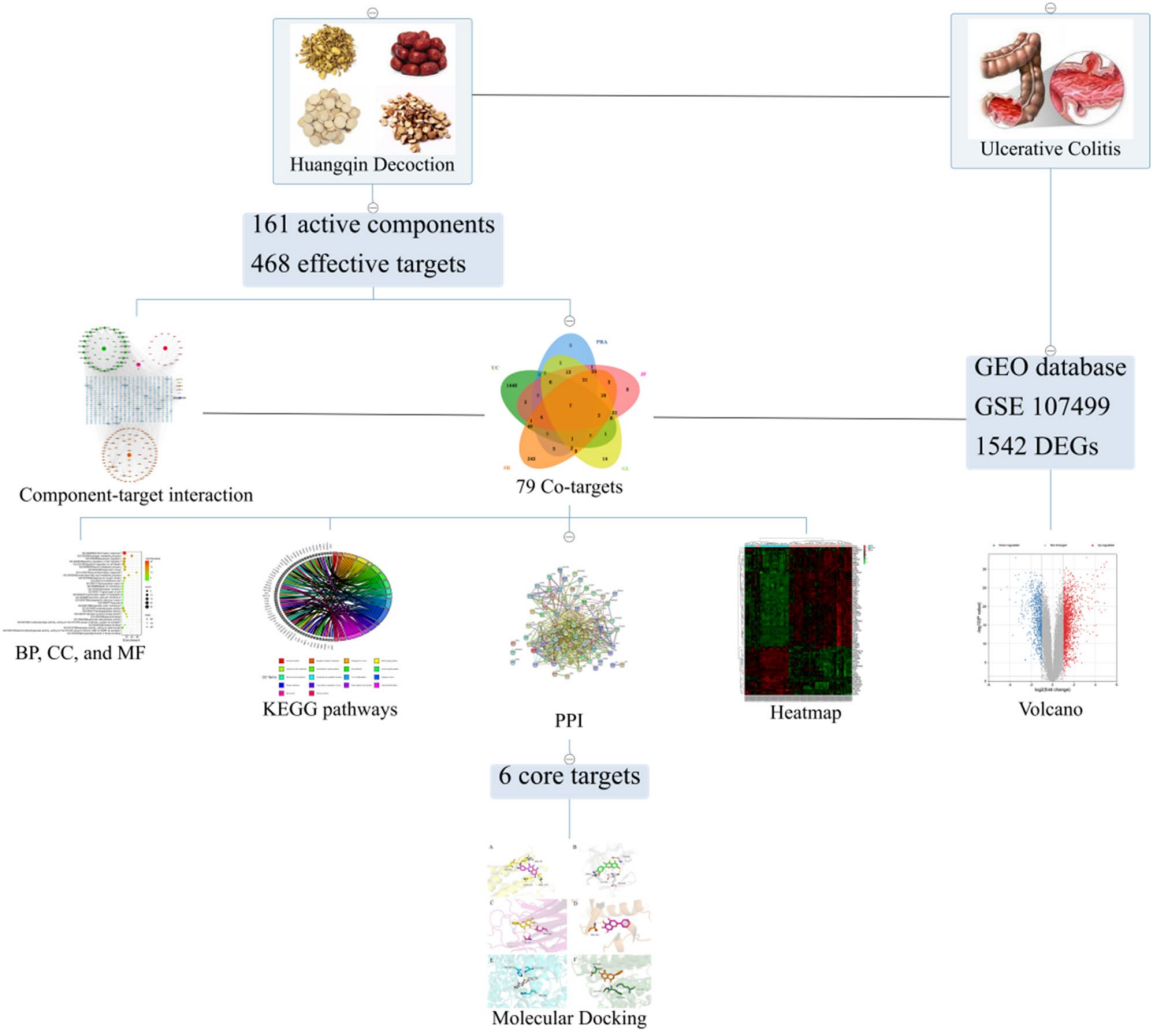
Workflow diagram of the network pharmacology combined bioinformatics analysis of HQD in the treatment of ulcerative colitis.

In this study, we combined network pharmacology and bioinformatics to reveal the core target and pathways that are participated in the pathogenesis of HQD against ulcerative colitis and screen for molecular targets of HQD for the treatment of ulcerative colitis. The workflow is shown in Fig. 1.

## Materials and methods

### Pharmacokinetic evaluation of active components

Traditional Chinese Medicine Systems Pharmacology Database and Analysis Platform (TCMSP, https://tcmspw.com/tcmsp.php^10^ were used to filter out active components, according to absorption, distribution, metabolism, excretion (ADME) criteria (OB ≥ 30% and DL ≥ 0.18)^11^. Confirmation of active components SMILES structures from PubChem (https://PubChem.ncbi.nlm.nih.gov) ^12^. Effective targets were selected from DrugBank (https://go.drugbank.com) and Swiss Target Prediction (http://www.swisstargetprediction.ch/). ^13^. Interaction of over 100 was recognized as an active component.

### Screening for DEGs of ulcerative colitis and analysis

Expression profiling data from GSE107499 (GPL15207 [PrimeView] Affymetrix Human Gene Expression Array) was obtained from the GEO database (http://www.ncbi.nlm.nih.gov/geo/) with 44 healthy individuals and 75 ulcerative colitis colonic mucosa samples. Based on the GEO2R web tool (http://www.ncbi.nlm.nih.gov/geo/geo2r/), differentially expressed genes (DEGs) with the cut-off criteria of |Log_2_FC|> 1 and adjusted *P*-value < 0.05 and visualized by volcano plot.

### Protein–protein interaction (PPI) network

Standardize gene names from Uniprot Knowledgebase (UniprotKB, http://www.uniprot.org), selecting “Homo sapiens”. Draw the Venn diagram (http://bioinformatics.psb.ugent.be/webtools/Venn/) for co-targets between HQD and DEGs and shown as a heatmap. Built on the co-targets result, the PPI network was conducted by Search Tool for the Retrieval of Interacting Genes (STRING, https://www.string-db.org/^14^, then visualized by Cytoscape3.7.2^15^. The core target was topological analyzed using the CytoNCA plugin, limited core targets over 1 time medians of degree, betweenness, and closeness, respectively.

### GO and KEGG pathway enrichment analysis

Metascape (http://metascape.org/^16^ serves as a gene annotation and analysis resource platform, limited “H.sapiens” and *P* < 0.01 for GO and KEGG pathway enrichment analysis^17^, selected top 10 results in bubble chart colored by − log10 (*P* values).

### Molecular docking

3D protein structure of core target was downloaded from RCSB Protein Data Bank (RCSB PDB, http://www.pdb.org/), using Pymol software to separate modified, ligand and remove water. 3D structure of core component was downloaded from PubChem, after adding hydrogen and calculate charges, then using Autodock Vina 1.1.2 software to dock with core targets, limited binding energy ≤ − 5.0 kcal·mol^-1^ as stable connection site.

## Results

### Identification of active components

**Figure 2:**
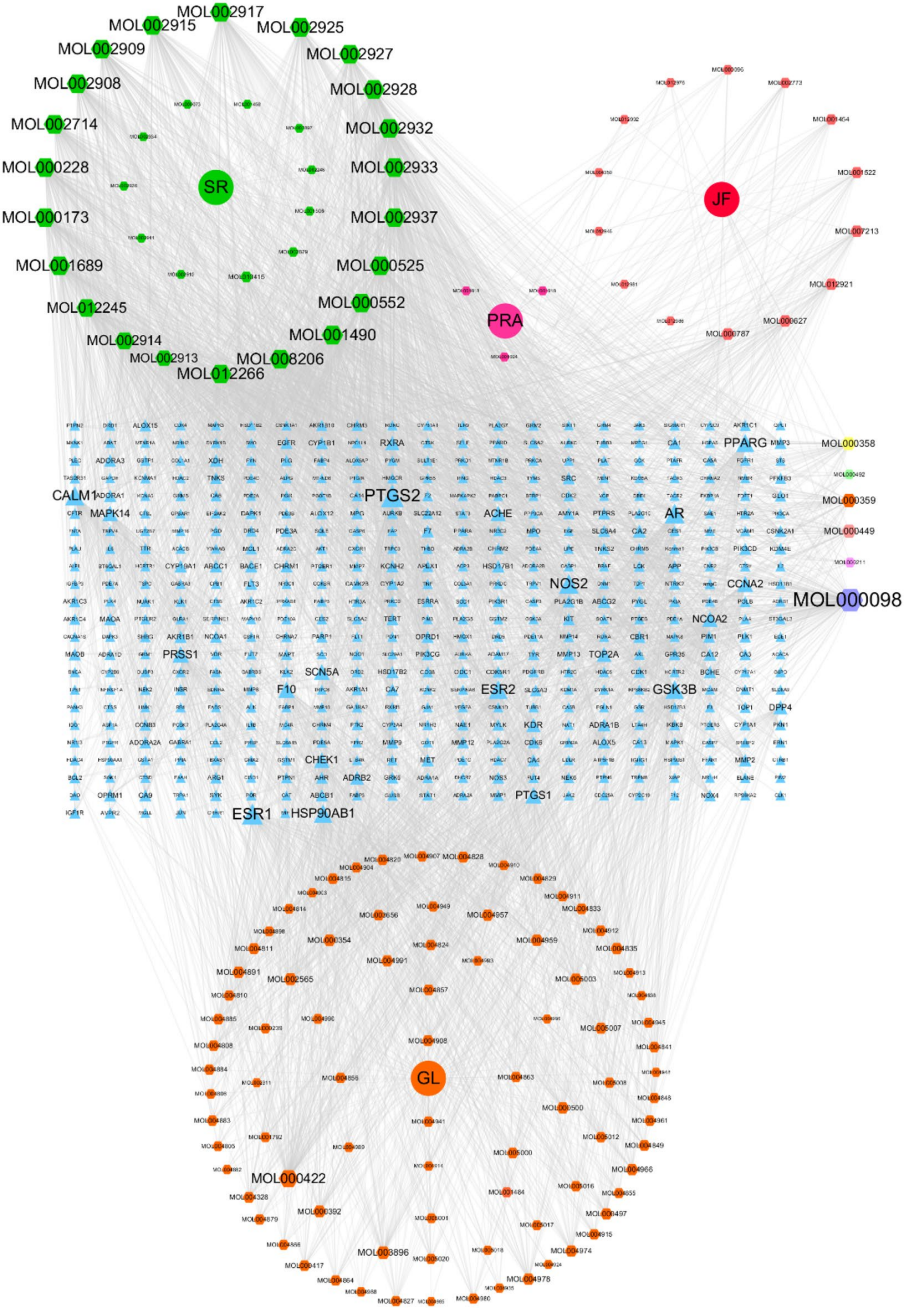
Active components-targets of HQD performed by Cytoscape3.7.2. Green nodes are SR components, pink nodes are PRA components, red nodes are JF components, orange nodes are GL components, blue nodes are drug targets. MOL000358 is in SP, PRA, JF; MOL000492 is in PRA and JF; MOL000359 is in SR, PRA, and GL; MOL000499 is in SR and JF; MOL000211 is in PRA, JF, and GL; MOL000098 is in JF and GL. The size represents the degree.

A total of 161 active components were obtained from the TCMSP database: 35 in *Scutellariae Radix* (SR, Huangqin), 13 in *Paeoniae Radix Alba* (PRA, Baishao), 29 in *Jujubae Fructus* (JF, Dazao), 92 in *Glycyrrhiza glabra L.* (GL, Gancao) (Fig. 2 and Supplement Table S1). Among 161 active components, 468 effective targets were selected by the Swiss Target Prediction and DrugBank database. Twenty core components as interactions over 100 were listed in Table 1.

**Table 1:**
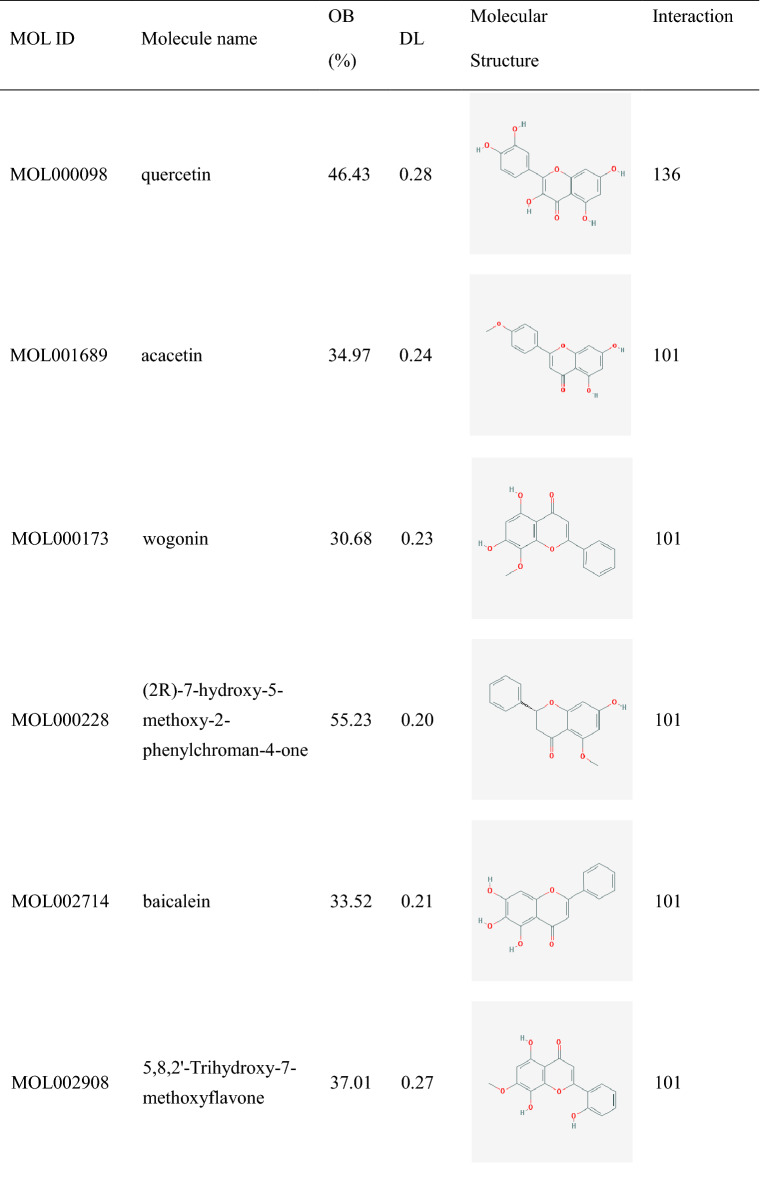

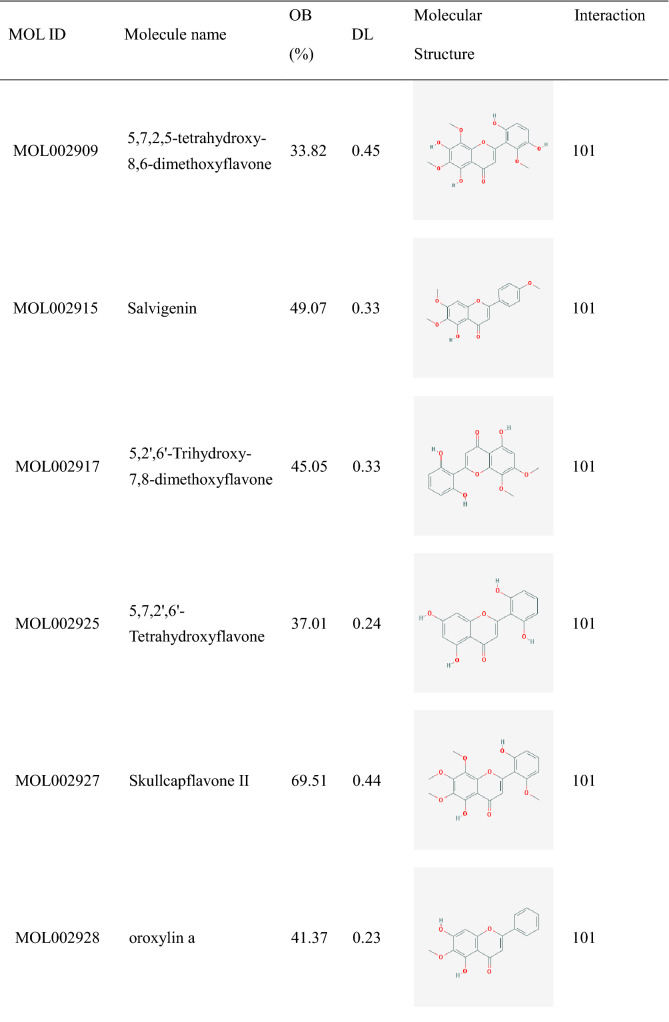

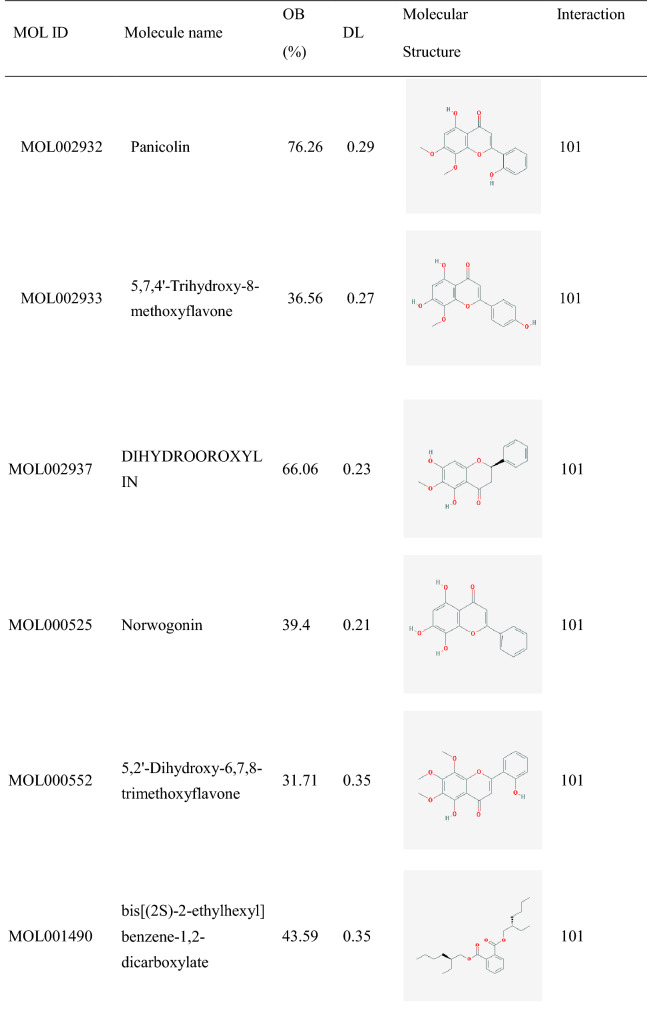

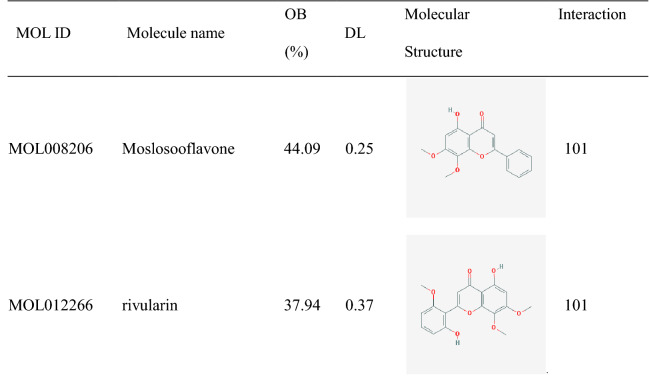
Twenty core active components of HQD.

### Identification of DEGs and co-targets

**Figure 3:**
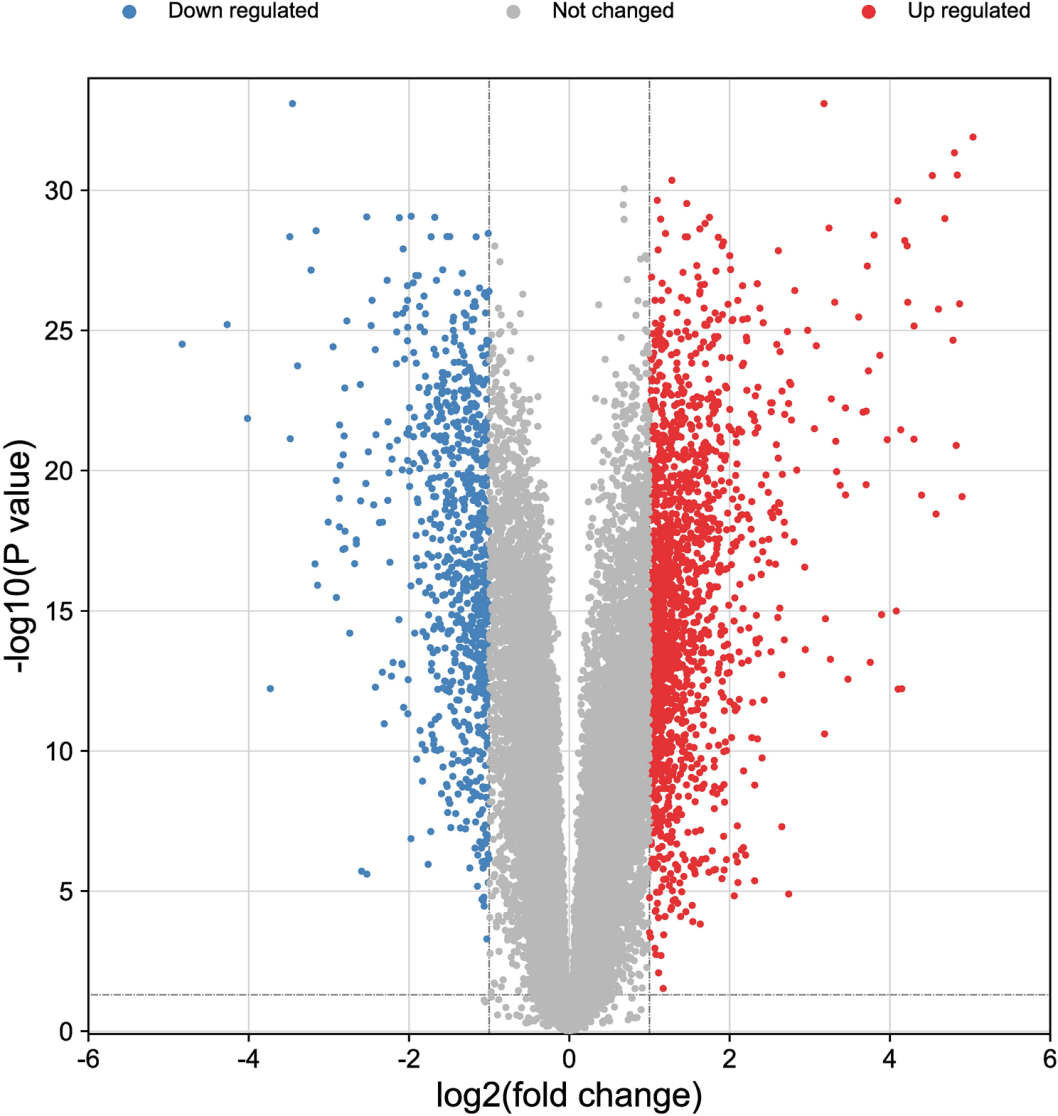
Volcano plot of DEGs. 1024 red dots are upregulated genes. 518 blue dots are downregulated genes. DEGs are defined with the cut-off criteria of |Log2FC|> 1 and adjusted *P*-value < 0.05.

**Figure 4:**
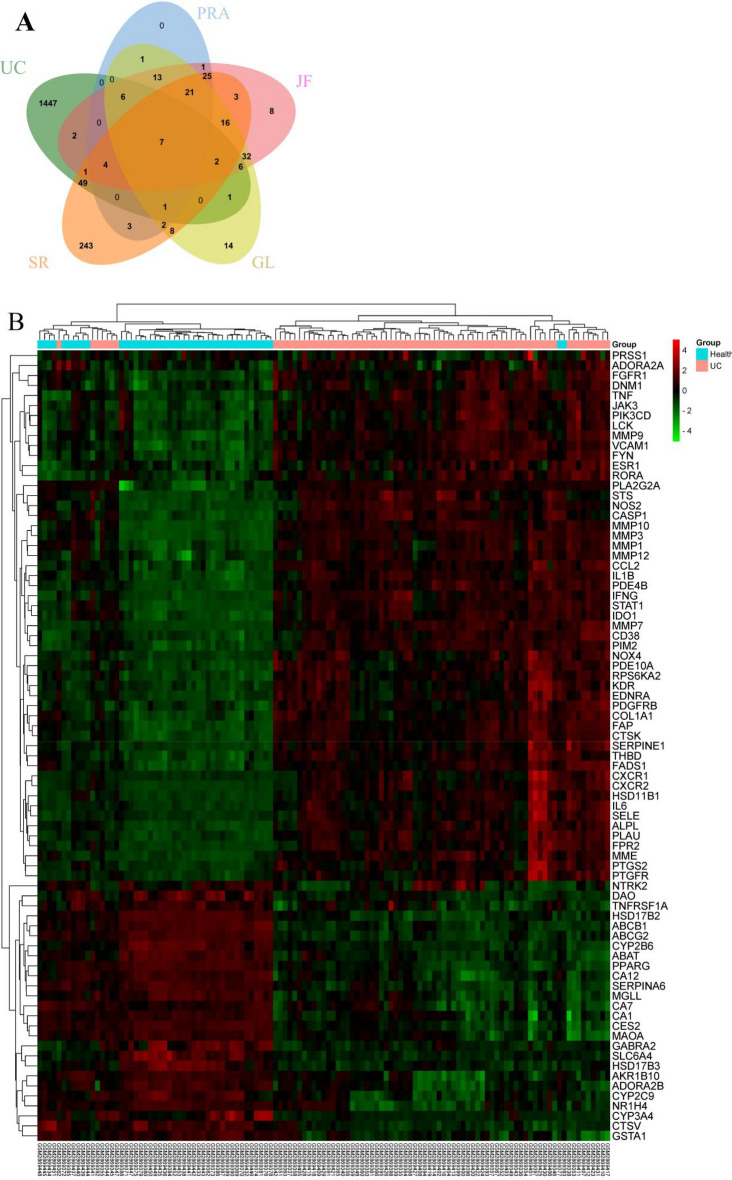
79 co-targets. (**A**) Venn analysis. The green is ulcerative colitis (1542 targets), blue is Paeoniae Radix Alba (84 targets), pink is Jujubae Fructus (147 targets), yellow is Glycyrrhiza glabra L. (130 targets), orange is Scutellariae Radix (385 targets). (**B**) Heatmap. Red dots are upregulated expression. Green dots are downregulated expression.

According to the GEO2R web tool, a total of 1542 DEGs were identified, which consist of 1024 upregulated and 518 downregulated genes (Fig. 3 and Supplement Table S2). Venn analysis between 1542 DEGs and 468 HQD targets, had got 79 co-targets (Fig. 4A). Hierarchical cluster analysis of 79 co-targets between ulcerative colitis mucosal samples and normal controls in GSE107499 (Fig. 4B).

### PPI network and core target

**Figure 5:**
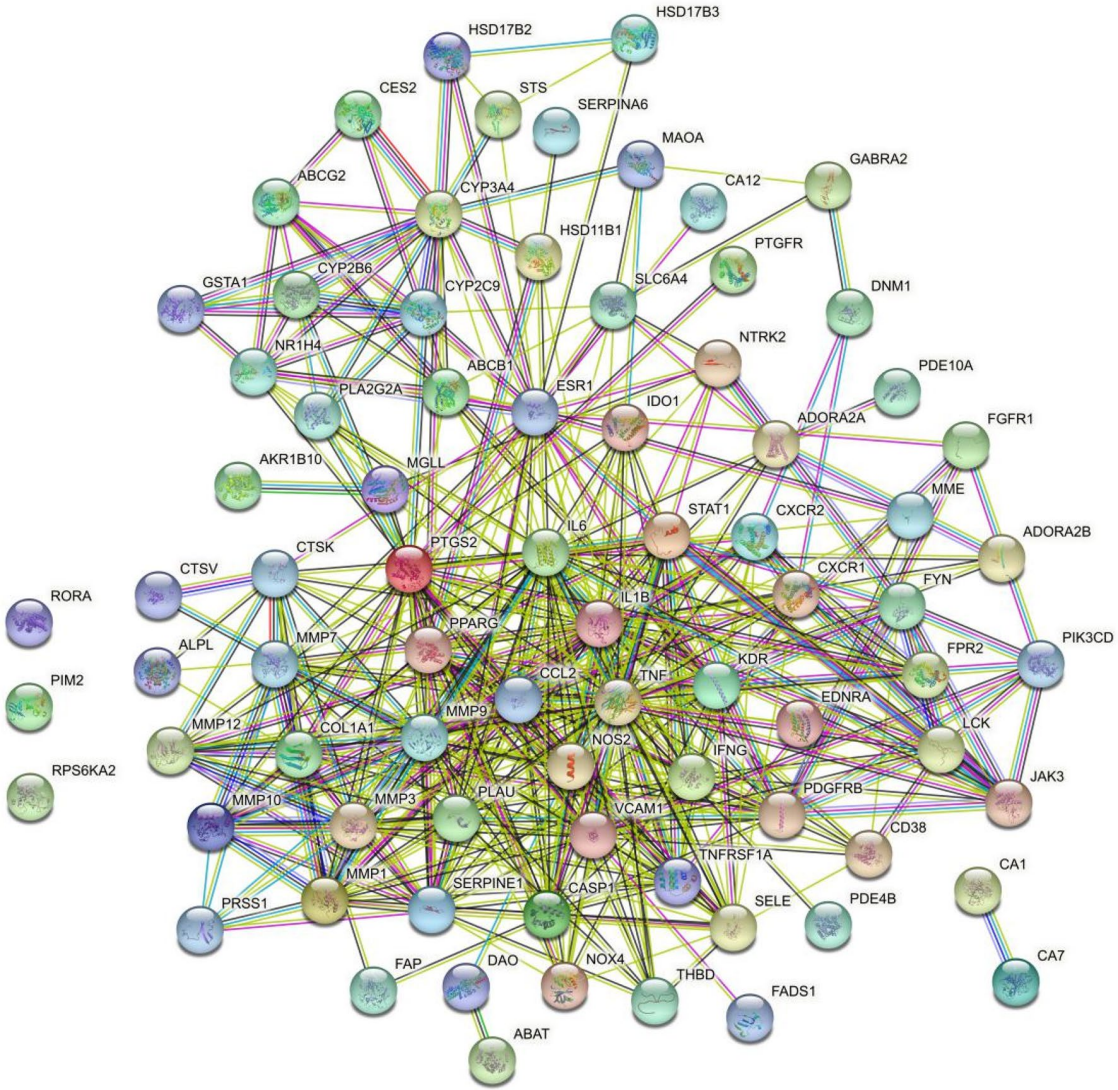
PPI network performed by STRING database. Contains 79 nodes with 449 edges. The average node degree is 11.4, avg. local clustering coefficient is 0.688, and PPI enrichment *P*-value < 1.0e−16.

In the PPI network (Fig. 5), after hiding 3 disconnected nodes (RORA, PIM2, RPS6KA2), 76 targets were chosen for topological analysis. According to the median degree (23), betweenness (342.25), and closeness (0.26) obtained by topological analysis, 6 core targets were further selected, including Interleukin-6 (IL6), Tumor necrosis factor (TNF), Interleukin-1 beta (IL1B), Prostaglandin G/H synthase 2 (PTGS2), Estrogen receptor (ESR1), Peroxisome proliferator-activated receptor gamma (PPARG). (Table 2 and Supplement Table S3).

**Table 2:** Topological analysis of PPI network.

Target	Degree	Betweenness	Closeness
IL6	45	684.4984	0.29411766
TNF	45	779.93317	0.29182878
IL1B	40	400.18173	0.28625953
PTGS2	33	523.231	0.2788104
ESR1	31	740.9644	0.27272728
PPARG	23	370.8105	0.2669039

### GO and KEGG pathway enrichment analysis

**Figure 6:**
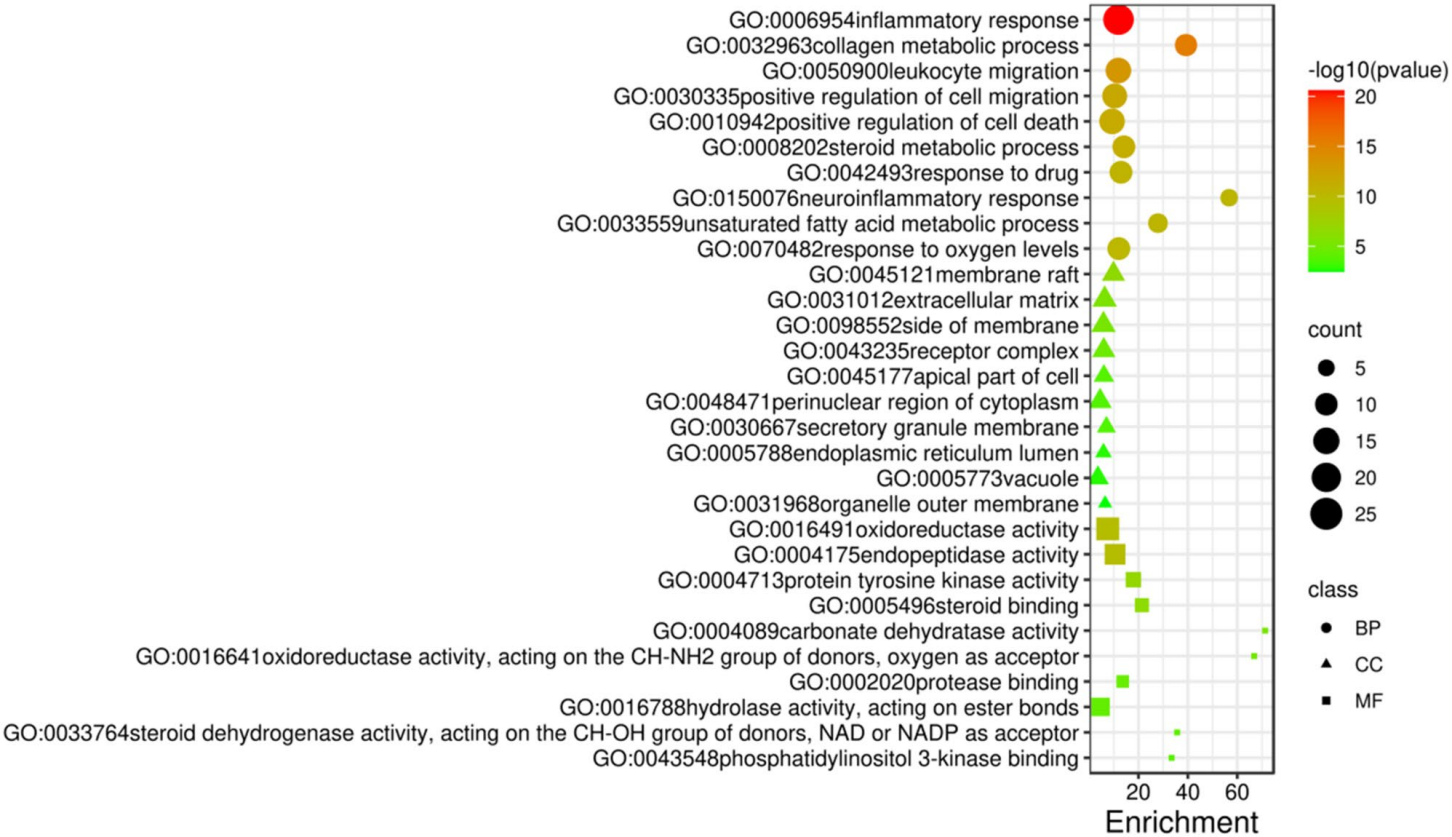
GO enrichment analysis of overlapping targets, including biological processes, cellular components, and molecular functions. The color represents the different − Log10 (*P* value), the size of the circle represents the counts.

The GO and KEGG pathway enrichment analysis of 79 overlapping targets were performed relying on Metascape platform according to the *P* < 0.01. There were 818, 26, and 66 GO terms related to biological processes, cell components, and molecular functions, respectively (Fig. 6). Inflammatory response, collagen metabolic process, and leukocyte migration was the significantly enriched for BP. For Membrane raft, extracellular matrix, and side of membrane was the significantly enriched for CC. Oxidoreductase activity, endopeptidase activity, and protein tyrosine kinase activity was the significantly enriched for MF.

**Figure 7:**
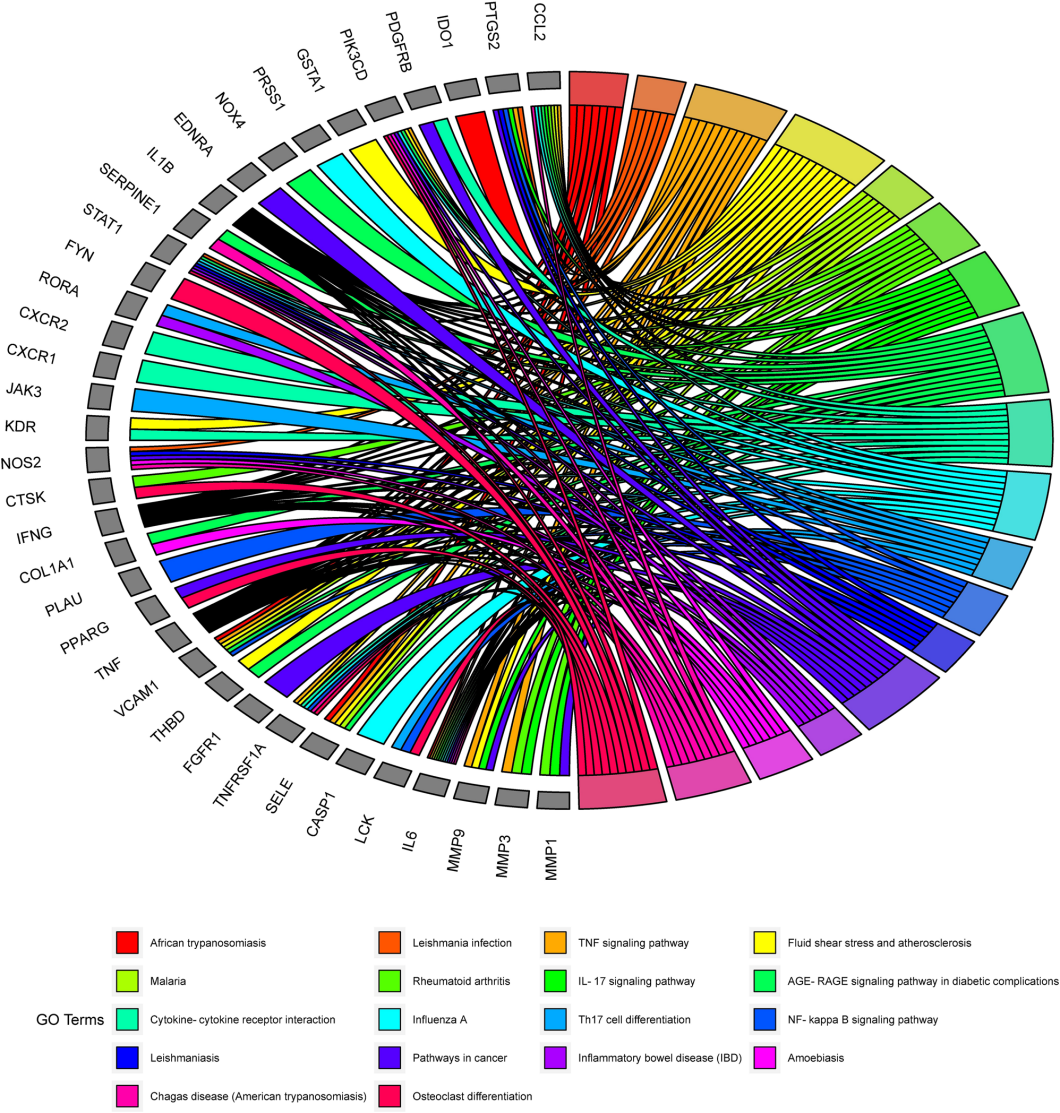
The top 20 pathways for KEGG enrichment analysis for overlapping 79 targets.

In addition, 184 pathways were contained in KEGG pathway enrichment analysis, mainly including TNF signaling pathway, IL-17 signaling pathway, Rheumatoid arthritis, Cytokine-cytokine receptor interaction, Th17 cell differentiation (Fig. 7).

### Molecular docking

Molecular docking was applied to analyse the binding of the core targets (IL6, TNF, IL1B, PTGS2,ESR1, and PPARG) and all 19 active compounds of HQD (except for MOL001490, 3D conformer generation is disallowed on PubChem since too flexible), respectively. The binding energy score was all smaller than − 5.0 kcal·mol^−1^, and the results are listed in Table 3.

**Table 3:** Molecular docking binding energy (kcal mol^−1^).

MOL ID	Molecular name	1ALU	1TNF	1L1B	5F19	3OS8	1PRG
MOL001689	acacetin	− 6.6	− 8.9	− 7.3	− 9.3	− 7.3	− 8.6
MOL000173	wogonin	− 6.3	− 8.6	− 7.3	− 9.0	− 8.5	− 8.6
MOL000228	(2R)-7-hydroxy-5-methoxy-2-phenylchroman-4-one	− 6.6	− 8.4	− 7.3	− 8.9	− 8.4	− 9.2
MOL002714	baicalein	− 7.0	− 9.2	− 7.1	− 9.3	− 8.4	− 9.3
MOL002908	5,8,2′-Trihydroxy-7-methoxyflavone	− 6.5	− 6.6	− 7.3	− 9.2	− 8.0	− 9.1
MOL002909	5,7,2,5-tetrahydroxy-8,6-dimethoxyflavone	− 6.1	− 9.1	− 7.1	− 8.8	− 7.6	− 7.8
MOL002915	Salvigenin	− 6.3	− 8.6	− 7.3	− 9.3	− 7.5	− 8.2
MOL002917	5,2′,6′-Trihydroxy-7,8-dimethoxyflavone	− 6.4	− 6.5	− 7.0	− 8.5	− 7.4	− 7.8
MOL002925	5,7,2′,6′-Tetrahydroxyflavone	− 6.7	− 9.0	− 7.3	− 9.2	− 8.2	− 8.2
MOL002927	Skullcapflavone II	− 6.0	− 6.3	− 7.1	− 8.1	− 7.6	− 7.6
MOL002928	oroxylin a	− 6.8	− 8.6	− 7.0	− 9.1	− 7.6	− 9.3
MOL002932	Panicolin	− 6.3	− 8.9	− 6.9	− 9.5	− 8.0	− 8.6
MOL002933	5,7,4′-Trihydroxy-8-methoxyflavone	− 6.4	− 8.8	− 7.2	− 8.8	− 7.2	− 9.0
MOL002937	DIHYDROOROXYLIN	− 6.7	− 8.7	− 7.0	− 8.9	− 7.5	− 9.2
MOL000525	Norwogonin	− 6.7	− 9.1	− 7.2	− 9.3	− 8.4	− 9.2
MOL000552	5,2′-Dihydroxy-6,7,8-trimethoxyflavone	− 6.5	− 6.2	− 7.0	− 9.5	− 7.2	− 8.6
MOL008206	Moslosooflavone	− 6.2	− 8.6	− 7.0	− 8.8	− 8.0	− 8.8
MOL012266	rivularin	− 6.0	− 6.5	− 7.0	− 8.0	− 7.5	− 7.8
MOL000098	Quercetin	− 7.2	− 9.1	− 7.6	− 9.4	− 8.3	− 9.1

**Figure 8:**
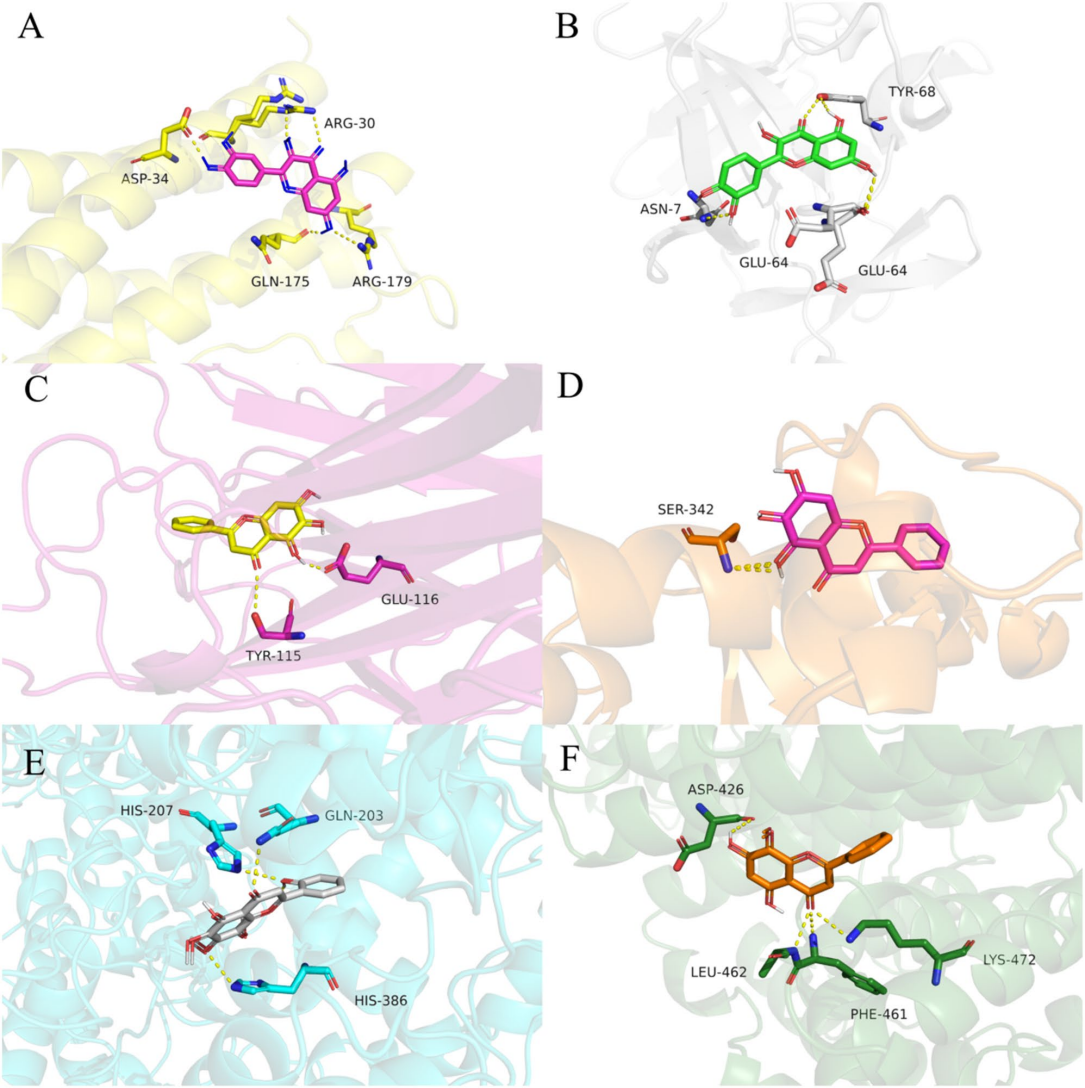
Molecular docking site. (**A**) IL6-quercetin. (**B**) IL1B-quercetin. (**C**)TNF-baicalein. (**D**)PPARG-baicalein. (**E**)PTGS2-5,2′-Dihydroxy-6,7,8-trimethoxyflavone. (**F**) ESR1-wogonin.

In detail, the active compound with the lowest binding energy to IL6 (RCSB PDB: 1ALU) and IL1B (RCSB PDB: 1I1B) was MOL000098 quercetin, TNF (RCSB PDB: 1TNF) and PPARG (RCSB PDB: 1PRG) was MOL002714 baicalein, PTGS2 (RCSB PDB: 5F19) was MOL000552 5,2′-Dihydroxy-6,7,8-trimethoxyflavone, and ESR1 (RCSB PDB: 3OS8) was MOL000173 wogonin. The binding energy scores of IL6-quercetin and IL1B-quercetin were − 7.2 and − 7.6 kcal·mol^-1^, TNF-baicalein and PPARG-baicalein were − 9.2 and − 9.3 kcal·mol^-1^, PTGS2-5,2′-Dihydroxy-6,7,8-trimethoxyflavone was − 9.5 kcal·mol^-1^, ESR1-wogonin was -8.5 kcal·mol^-1^, respectively (Fig. 8). These consequences state that the screened core targets indeed stably combine with HQD.

Furthermore, according to the ZINC15 database, quercetin (ZINC4175638), baicalein (ZINC3871633), and wogonin (ZINC899093) as the stable compound recognized as key compounds. From Table 3, the binding energy of 5F19 and 1PRG was lower than others, which indicated that PTGS2 and PPARG were the target of HQD on ulcerative colitis.

## Discussion

Given its unclear pathogenesis, potentially progressive, and debilitating disease course, the therapeutic goals for ulcerative colitis have changed from treating symptoms to mucosal healing. However, conventional treatments are mainly restricted to control inflammation and clinical symptoms, and hard to meet the expectations of long-term management. Therefore, combination therapy is the urgent strategy for ulcerative colitis^18^. Profound knowledge of genetics, microbiota or cytokine imbalances in the bowel and its function in inflammation will sustain the outcome of possible therapeutic approaches if we act on those targets. In addition, the development of new ligands with therapeutic efficacy on the bowel would be a potential avenue in the search for treatments or prevention of ulcerative colitis. To date, with widely using TCM in different diseases, clinical effectiveness has been demonstrated. As for this concern, we made a combination of network pharmacology and bioinformatics might contribute to a further understanding of the disease aetiology and find novel potential therapeutic way.

In this study, 20 core components with 486 targets of HQD and 1542 DEGs (GSE107499) of ulcerative colitis were examined. 79 co-targets which indicated that the targets of HQD against ulcerative colitis may be through IL6, TNF, IL1B, PTGS2, ESR1,PPARG, which have a high degree in PPI network. IL6, TNF, IL1B have been proved that have a strong impact on the pathological process, as for docking test, we found PTGS2 and PPARG have the lower energy than others, and we focus on more details in PTGS2, ESR1, and PPARG.

ESR1, a nuclear hormone receptor, is involved in the regulation of eukaryotic gene expression and affects cellular proliferation and differentiation in target tissues by the steroid hormones and their receptors. ESR1 has mutual transrepression with NF-κB, e.g. decreases NF-κB DNA-binding activity and inhibits NF-κB-mediated transcription^19^. Also, it can involve activation of NOS3 and endothelial nitric oxide production^20^. Dysregulation of estrogen receptors in the intestinal mucosa of ulcerative colitis indicates that estrogen signaling may play a role in the local immune response and maintain epithelial homeostasis in a gender- and age-dependent manner^21^, even though there is no evidence in difference prevalence in male and female.

PPARG (PPARγ), a new coming experiment targeting the treatment of ulcerative colitis, is one of the isoforms of nuclear PPAR receptors that belong to the steroid receptor superfamily. It can be bound to DNA-specific PPAR response elements (PPRE) and modulates the transcription of its target genes, so that controls the peroxisomal beta-oxidation pathway of fatty acids. In addition to playing a role in the regulation of cardiovascular circadian rhythms adipocyte differentiation, and glucose homeostasis, PPARγ also acts as a critical regulator of gut homeostasis by suppressing NF-κB-mediated proinflammatory responses^22–24^.

Presence of PPARγ can be observed throughout the gastrointestinal tract: mesenteric adipocytes, macrophages, and epithelium, most prevalently in the more differentiated layer of epithelial cell, have the highest levels of PPARγ mRNA than other tissue^25–28^. However, disease onset of ulcerative colitis provokes inflammation of the colonic intestinal wall, showing significant downregulation of PPARγ, compared with remission mucosal tissue^29–31^. Increased transcriptional activity of PPARγ was linked to decreased intestinal inflammation in DSS-induced colitis mice^32^.

Besides, a present study has proved that PPARγ can be activation by COX-2 products (15d-PGJ2-G, a chemical metabolite of PGD2-G) to perform anti-inflammatory in ulcerative colitis^33^. PTGS2 (COX-2) is a key gene with a particular role in the inflammatory response and expresses in epithelial cells and mononuclear cells in IBD^34,35^. Prostaglandins are critical to the process of mucosal healing in the colonic tract^36^.

Moreover, core components of HQD act in a multi-component and multi-target way. Quercetin, acacetin, wogonin, norwogonin, baicalein, salvigenin, oroxylin a were key active components of HQD in the treatment of ulcerative colitis. Quercetin has an antioxidant effect, suppresses proinflammatory mediators' release and expression of inflammatory proteins^37^. Acacetin by inhibiting inflammation and regulating the intestinal microbiota in DSS-induced colitis mice^38^. Wogonin is quickly converted into baicalin and various flavones in metabolism and may act in the PI3K-Akt pathway, AMPK pathways, and inhibition of telomerase activity^39^. Baicalin has been confirmed to inhibit IL-33 and NF-κB p65 expression, increase IκB-α levels, regulate Th17/Treg balance and gut microbiota to act antiinflammation^40,41^. Oroxylin A as the main component extracted from *Scutellariae radix* can decrease the expression of inflammatory cytokines IL-6 and IL-1β, and attenuated IL-6/STAT3 signaling pathway in ulcerative colitis mice^42^. Norwogonin can increase Bax and decrease in Bcl-2 levels, induction of autophagy along with triggering G2/M phase cell cycle arrest to suppress the colon cancer^43^.

In GO and KEGG pathway enrichment analysis, HQD may through several cellular components, such as membrane raft, extracellular matrix, and receptor complex to active in molecular functions, including oxidoreductase activity, endopeptidase activity, and protein kinase activity to get involved in biological processes, such as TNF signaling pathway, IL-17 signaling pathway, Th17 cell differentiation, and NF-kappa B signaling pathway.

Recover intestinal mucosal homeostasis faces a particularly challenging change, because it is against infection, commensal organisms, and unbalance T cells differentiation^44^. Th17 is the most synthetic T cell, plays a vital role in gut between homeostasis and inflammation, and is thought to play a pathogenic role in IBD^45,46^. In a previous study, Th17 cell differentiation can be driven by lnc-ITSN1-2 and correlate with increased IBD risk, activity, and inflammatory cytokines^47^. NF-κB signaling is often dysregulated resulting in overzealous inflammation and distinctly into two pathways, canonical or non-canonical^48^. Different from rapid, acute inflammation is canonical NF-κB signaling pathway, non-canonical NF-κB signaling has become more evident in maintaining immune system homeostasis in the gut, which regulates T cells differentiation and function^49,50^. In sum, HQD mainly acts into multi-component, multi-target, multi-pathway in treating ulcerative colitis.

## Limitations

Further pharmacological experiments are needed in order to validate the therapeutic mechanism of HQD on ulcerative colitis.

## Conclusion

In this study, we combined bioinformatics analysis, network pharmacology, and molecular docking to reveal the mechanisms of HQD against ulcerative colitis. HQD may be through multi-components and multi-pathway in treating ulcerative colitis.

## Supplementary Information

Supplementary Tables.

## Data Availability

The data used to support the findings of this study is available.

## Abbreviations

TCM: Traditional Chinese Medicine
UC: Ulcerative colitis
IBD: Inflammatory bowel disease
HQD: Huangqin decoction
SR: *Scutellariae Radix*
PRA: *Paeoniae Radix Alba*
JF: *Jujubae Fructus*
GL: *Glycyrrhiza glabra L.*
TCMSP: Traditional Chinese Medicine Systems Pharmacology Database and Analysis Platform
UniprotKB: Uniprot knowledgebase
GEO: Gene expression omnibus
DEGs: Differentially expressed genes
STRING: Search Tool for the retrieval of interacting genes
PPI: Protein–protein interaction
GO: Gene Ontology
KEGG: The Kyoto encyclopedia of genes and genomes
IL6: Interleukin-6
TNF: Tumor necrosis factor
IL1B: Interleukin-1 beta
PTGS2: Prostaglandin G/H synthase 2
ESR1: Estrogen receptor
PPARG: Peroxisome proliferator-activated receptor gamma
