# Supplementary material for: Integrated bioinformatics and network pharmacology to identify the therapeutic target and molecular mechanisms of Huangqin decoction on ulcerative Colitis
Source: Sci Rep. 2022 Jan 7;12:159. doi: 10.1038/s41598-021-03980-8 (PMC8741777; doi:10.1038/s41598-021-03980-8)
Supplement: Supplementary file 1 — Supplementary Tables. [file 41598_2021_3980_MOESM1_ESM.docx]

Integrated bioinformatics and network pharmacology to identify the therapeutic target and molecular mechanisms of Huangqin Decoction on Ulcerative Colitis

Yi Wu^a,b,*^ Xinqiao Liu^a^ Guiwei Li ^a^

*^a^ First Teaching Hospital of Tianjin University of Traditional Chinese Medicine Tianjin 300000*

*^b^ National Clinical Research Center for Chinese Medicine Acupuncture and Moxibustion Tianjin 300000*

**Corresponding author: Yi Wu*

1. *mail：wuyi_96@qq.com*

*Phone: +86 18202246621*

**Supplementary tables:**

**Table S1.** Active components of HQD

| MOL ID | Molecule name | OB  (%) | DL | Molecular  Structure | Herb |
| --- | --- | --- | --- | --- | --- |
| MOL001689 | acacetin | 34.97 | 0.24 | 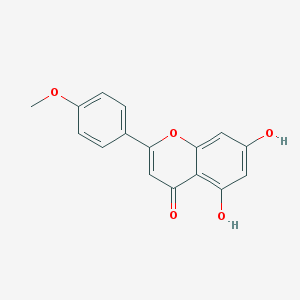 | SR |
| MOL000173 | wogonin | 30.68 | 0.23 | 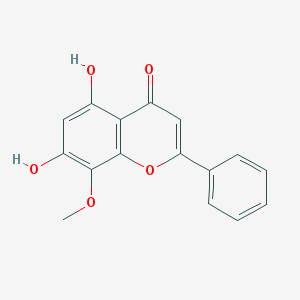 | SR |
| MOL000228 | (2R)-7-hydroxy-5-methoxy-2-phenylchroman-4-one | 55.23 | 0.2 | 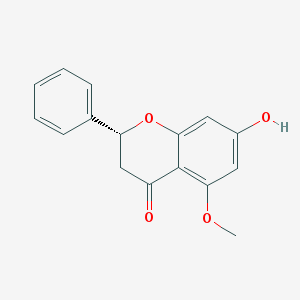 | SR |
| MOL002714 | baicalein | 33.52 | 0.21 | 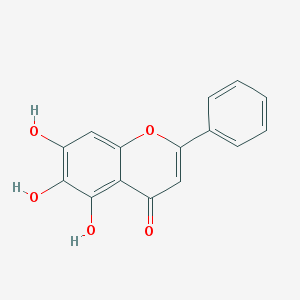 | SR |
| MOL002908 | 5,8,2'-Trihydroxy-7-methoxyflavone | 37.01 | 0.27 | 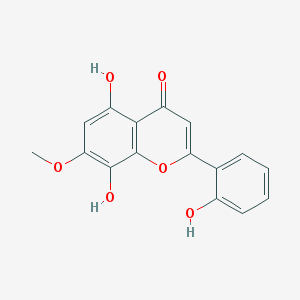 | SR |
| MOL002909 | 5,7,2,5-tetrahydroxy-8,6-dimethoxyflavone | 33.82 | 0.45 | 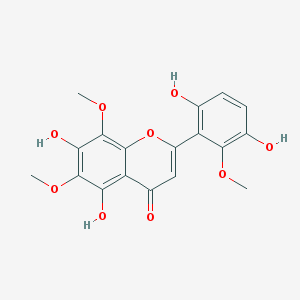 | SR |
| MOL002910 | Carthamidin | 41.15 | 0.24 | 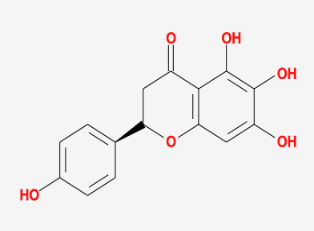 | SR |
| MOL002911 | 2,6,2',4'-tetrahydroxy-6'-methoxychaleone | 69.04 | 0.22 | 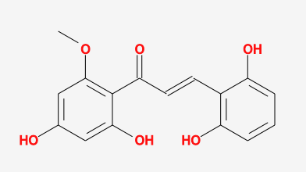 | SR |
| MOL002913 | Dihydrobaicalin_qt | 40.04 | 0.21 | 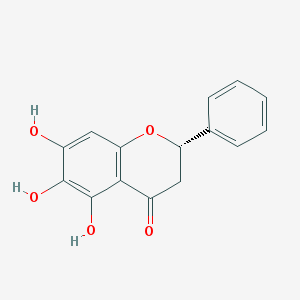 | SR |
| MOL002914 | Eriodyctiol (flavanone) | 41.35 | 0.24 | 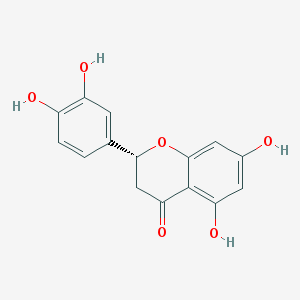 | SR |
| MOL002915 | Salvigenin | 49.07 | 0.33 | 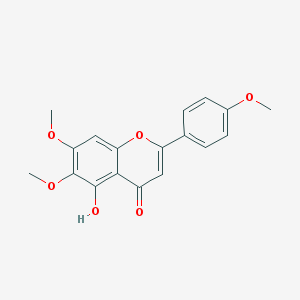 | SR |
| MOL002917 | 5,2',6'-Trihydroxy-7,8-dimethoxyflavone | 45.05 | 0.33 | 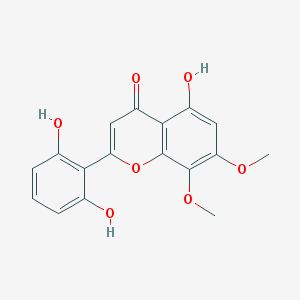 | SR |
| MOL002925 | 5,7,2',6'-Tetrahydroxyflavone | 37.01 | 0.24 | 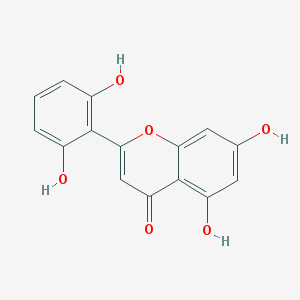 | SR |
| MOL002926 | dihydrooroxylin A | 38.72 | 0.23 | 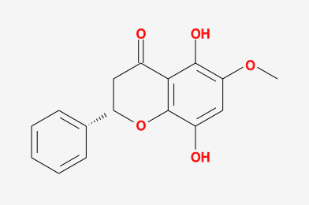 | SR |
| MOL002927 | Skullcapflavone II | 69.51 | 0.44 | 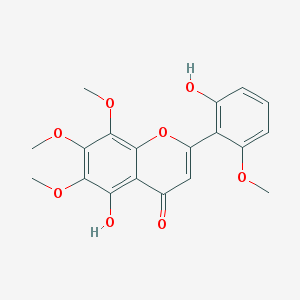 | SR |
| MOL002928 | oroxylin a | 41.37 | 0.23 | 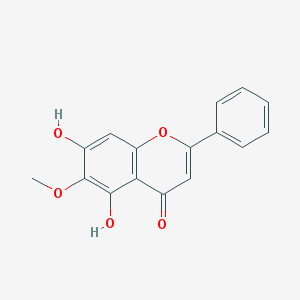 | SR |
| MOL002932 | Panicolin | 76.26 | 0.29 | 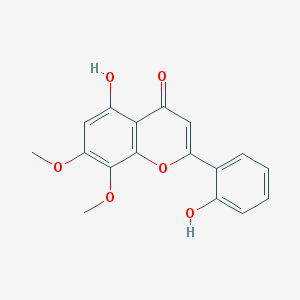 | SR |
| MOL002933 | 5,7,4'-Trihydroxy-8-methoxyflavone | 36.56 | 0.27 | 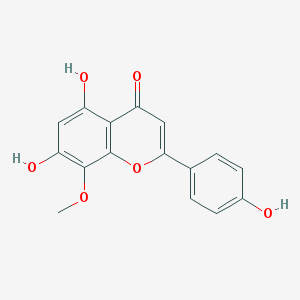 | SR |
| MOL002934 | NEOBAICALEIN | 104.34 | 0.44 | 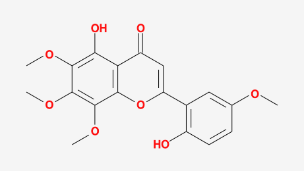 | SR |
| MOL002937 | DIHYDROOROXYLIN | 66.06 | 0.23 | 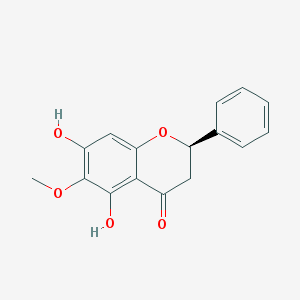 | SR |
| MOL000358 | beta-sitosterol | 36.91 | 0.75 | 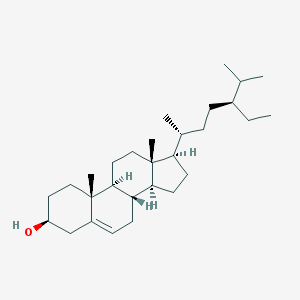 | SR,  PRA,  JF |
| MOL000359 | sitosterol | 36.91 | 0.75 | 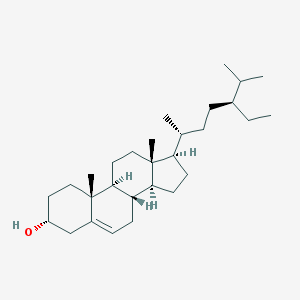 | SR,  PRA,  GL |
| MOL000525 | Norwogonin | 39.4 | 0.21 | 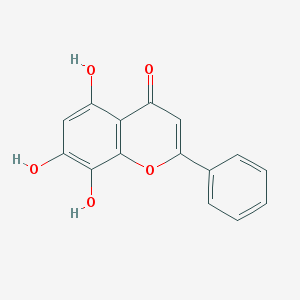 | SR |
| MOL000552 | 5,2'-Dihydroxy-6,7,8-trimethoxyflavone | 31.71 | 0.35 | 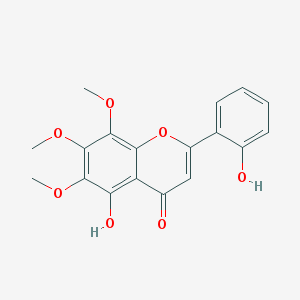 | SR |
| MOL000073 | ent-Epicatechin | 48.96 | 0.24 | 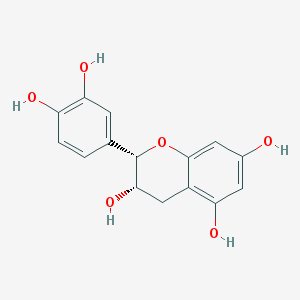 | SR |
| MOL000449 | Stigmasterol | 43.83 | 0.76 | 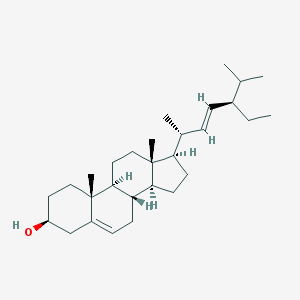 | SR,  JF |
| MOL001458 | coptisine | 30.67 | 0.86 | 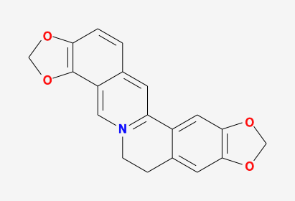 | SR |
| MOL001490 | bis[(2S)-2-ethylhexyl] benzene-1,2-dicarboxylate | 43.59 | 0.35 | 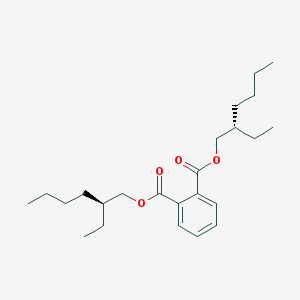 | SR |
| MOL001506 | Supraene | 33.55 | 0.42 | 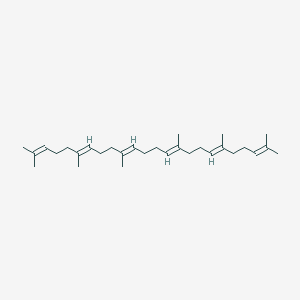 | SR |
| MOL002879 | Diop | 43.59 | 0.39 | 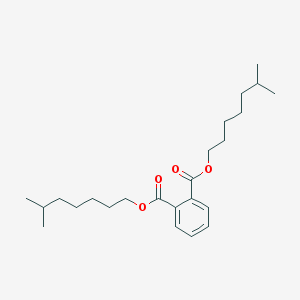 | SR |
| MOL002897 | epiberberine | 43.09 | 0.78 | 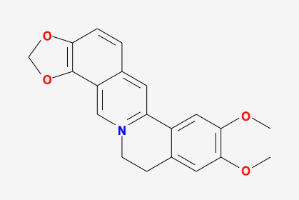 | SR |
| MOL008206 | Moslosooflavone | 44.09 | 0.25 | 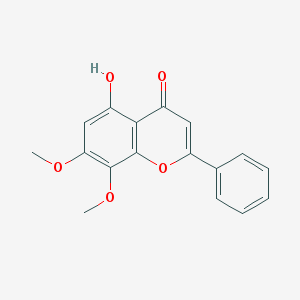 | SR |
| MOL010415 | 11,13-Eicosadienoic acid, methyl ester | 39.28 | 0.23 | 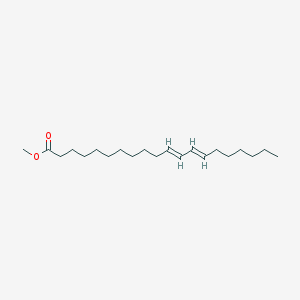 | SR |
| MOL012245 | 5,7,4'-trihydroxy-6-methoxyflavanone | 36.63 | 0.27 | 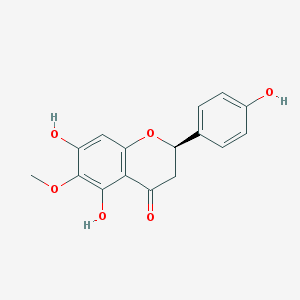 | SR |
| MOL012246 | 5,7,4'-trihydroxy-8-methoxyflavanone | 74.24 | 0.26 | 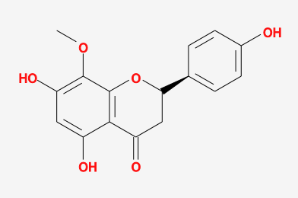 | SR |
| MOL012266 | rivularin | 37.94 | 0.37 | 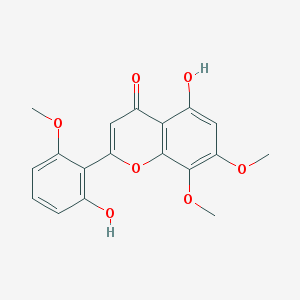 | SR |
| MOL001910 | 11alpha,12alpha-epoxy-3beta-23-dihydroxy-30-norolean-20-en-28,12beta-olide | 64.77 | 0.38 | 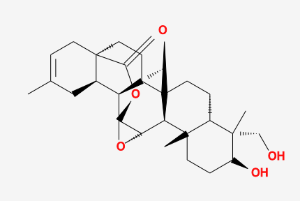 | PRA |
| MOL001918 | paeoniflorgenone | 87.59 | 0.37 | 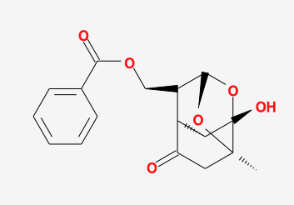 | PRA |
| MOL001919 | (3S,5R,8R,9R,10S,14S)-3,17-dihydroxy-4,4,8,10,14-pentamethyl-2,3,5,6,7,9-hexahydro-1H-cyclopenta[a]phenanthrene-15,16-dione | 43.56 | 0.53 | 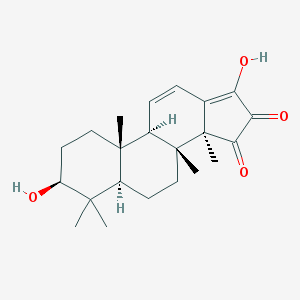 | PRA |
| MOL001921 | Lactiflorin | 49.12 | 0.8 | 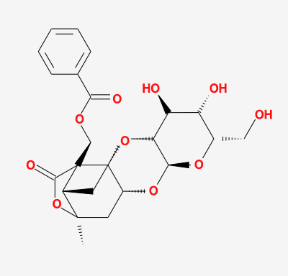 | PRA |
| MOL001924 | paeoniflorin | 53.87 | 0.79 | 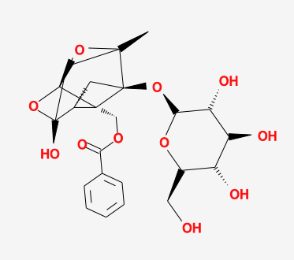 | PRA |
| MOL001925 | paeoniflorin_qt | 68.18 | 0.4 | 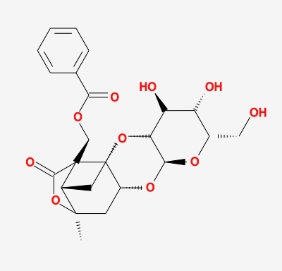 | PRA |
| MOL001928 | albiflorin_qt | 66.64 | 0.33 | 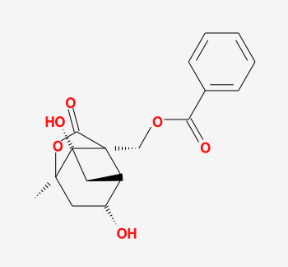 | PRA |
| MOL001930 | benzoyl paeoniflorin | 31.27 | 0.75 | 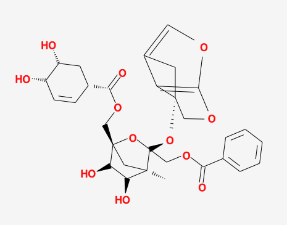 | PRA |
| MOL000211 | Mairin | 55.38 | 0.78 | 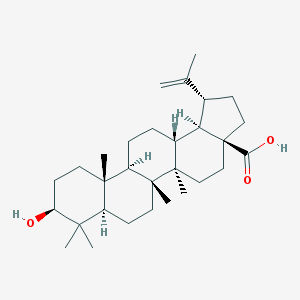 | PRA,  JF,  GL |
| MOL000422 | kaempferol | 41.88 | 0.24 | 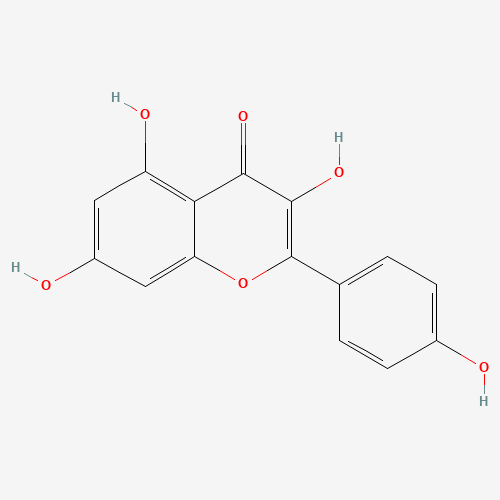 | PRA,  GL |
| MOL000492 | (+)-catechin | 54.83 | 0.24 | 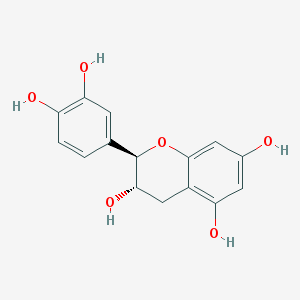 | PRA,  JF |
| MOL012921 | stepharine | 31.55 | 0.33 | 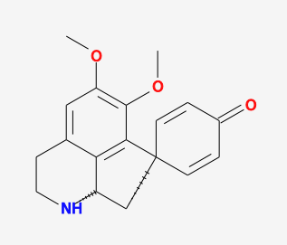 | JF |
| MOL012940 | Spiradine A | 113.52 | 0.61 | 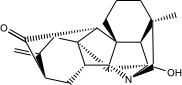 | JF |
| MOL012946 | zizyphus saponin I_qt | 32.69 | 0.62 | 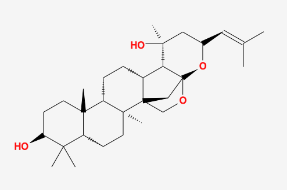 | JF |
| MOL012961 | jujuboside A_qt | 36.67 | 0.62 | 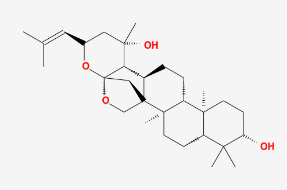 | JF |
| MOL012976 | coumestrol | 32.49 | 0.34 | 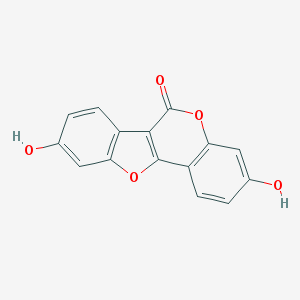 | JF |
| MOL012980 | Daechuine S6 | 46.48 | 0.79 | 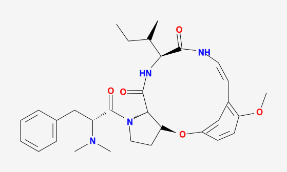 | JF |
| MOL012981 | Daechuine S7 | 44.82 | 0.83 | 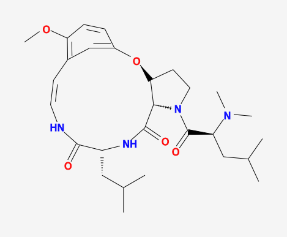 | JF |
| MOL012986 | Jujubasaponin V_qt | 36.99 | 0.63 | 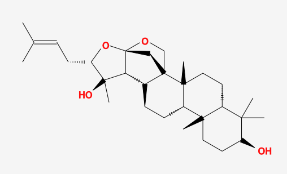 | JF |
| MOL012989 | Jujuboside C_qt | 40.26 | 0.62 | 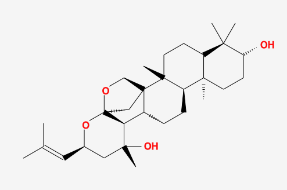 | JF |
| MOL012992 | Mauritine D | 89.13 | 0.45 | 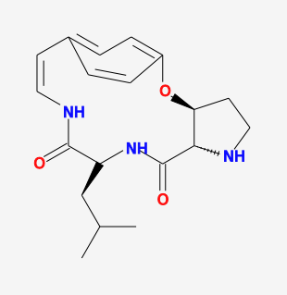 | JF |
| MOL001454 | berberine | 36.86 | 0.78 | 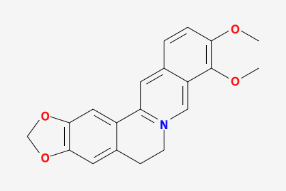 | JF |
| MOL001522 | (S)-Coclaurine | 42.35 | 0.24 | 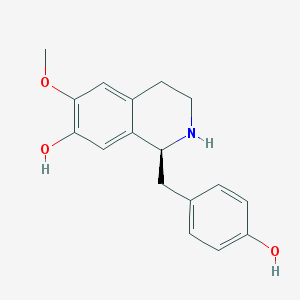 | JF |
| MOL003410 | Ziziphin_qt | 66.95 | 0.62 | 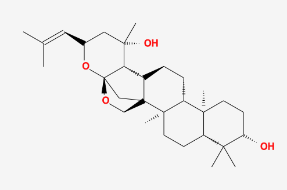 | JF |
| MOL004350 | Ruvoside_qt | 36.12 | 0.76 | 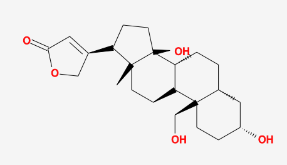 | JF |
| MOL000492 | (+)-catechin | 54.83 | 0.24 | 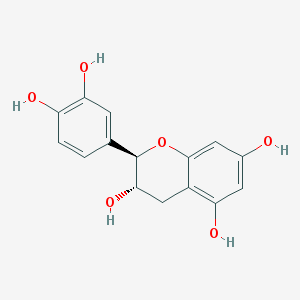 | JF |
| MOL005360 | malkangunin | 57.71 | 0.63 | 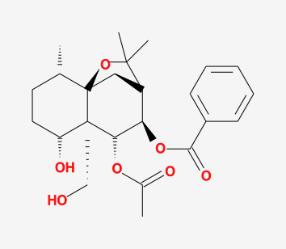 | JF |
| MOL000627 | Stepholidine | 33.11 | 0.54 | 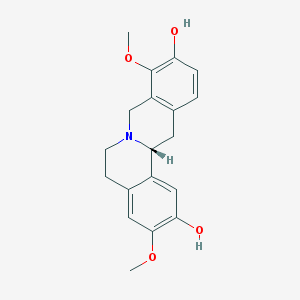 | JF |
| MOL007213 | Nuciferin | 34.43 | 0.4 | 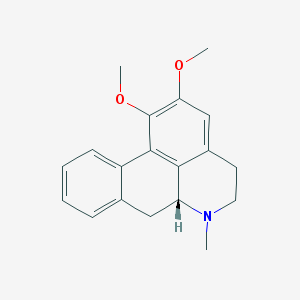 | JF |
| MOL000783 | Protoporphyrin | 30.86 | 0.56 | 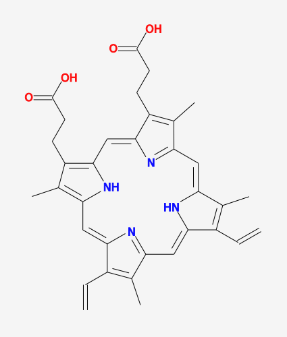 | JF |
| MOL000787 | Fumarine | 59.26 | 0.83 | 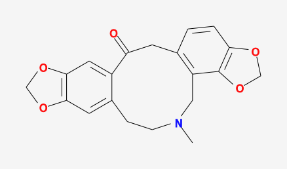 | JF |
| MOL008034 | 21302-79-4 | 73.52 | 0.77 | 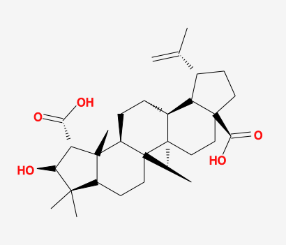 | JF |
| MOL008647 | Moupinamide | 86.71 | 0.26 | 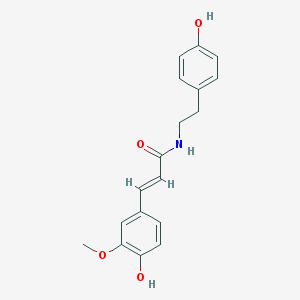 | JF |
| MOL002773 | beta-carotene | 37.18 | 0.58 | 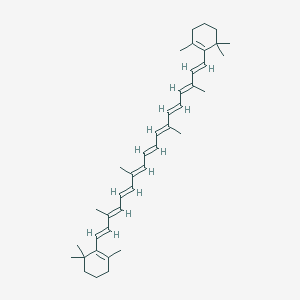 | JF |
| MOL000096 | (-)-catechin | 49.68 | 0.24 | 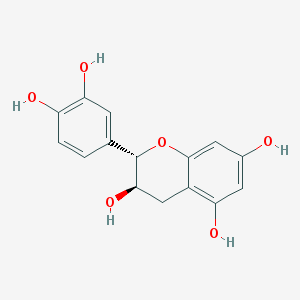 | JF |
| MOL000098 | quercetin | 46.43 | 0.28 | 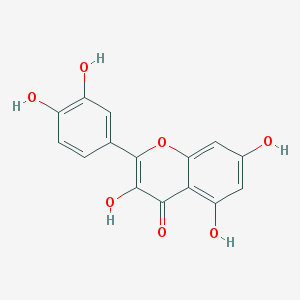 | JF,  GL |
| MOL013357 | (3S,6R,8S,9S,10R,13R,14S,17R)-17-[(1R,4R)-4-ethyl-1,5-dimethylhexyl]-10,13-dimethyl-2,3,6,7,8,9,11,12,14,15,16,17-dodecahydro-1H-cyclopenta[a]phenanthrene-3,6-diol | 34.37 | 0.78 | 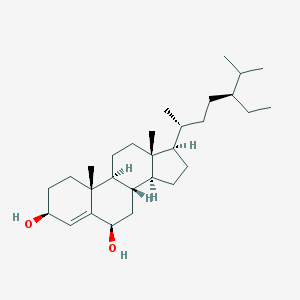 | JF |
| MOL001484 | Inermine | 75.18 | 0.54 | 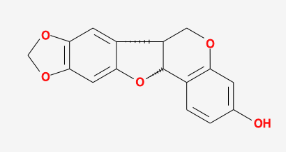 | GL |
| MOL001792 | DFV | 32.76 | 0.18 | 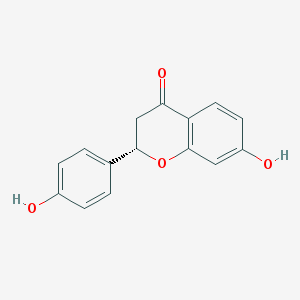 | GL |
| MOL002311 | Glycyrol | 90.78 | 0.67 | 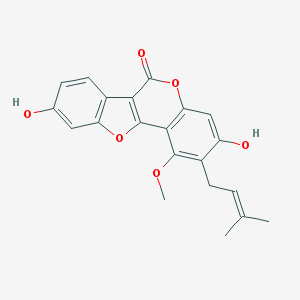 | GL |
| MOL000239 | Jaranol | 50.83 | 0.29 | 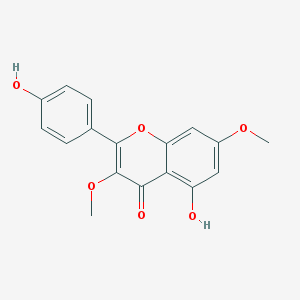 | GL |
| MOL002565 | Medicarpin | 49.22 | 0.34 | 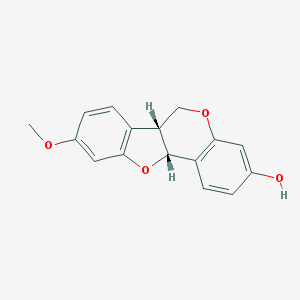 | GL |
| MOL000354 | isorhamnetin | 49.6 | 0.31 | 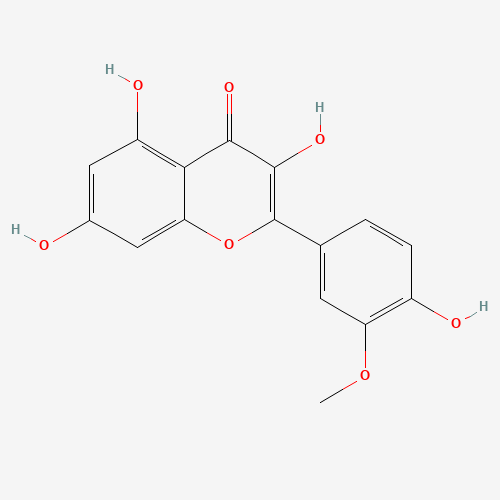 | GL |
| MOL003656 | Lupiwighteone | 51.64 | 0.37 | 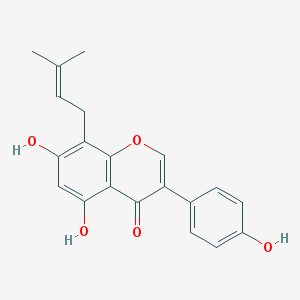 | GL |
| MOL003896 | 7-Methoxy-2-methyl isoflavone | 42.56 | 0.2 | 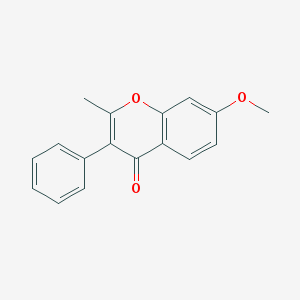 | GL |
| MOL000392 | formononetin | 69.67 | 0.21 | 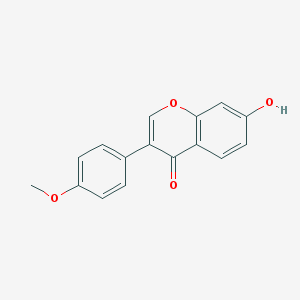 | GL |
| MOL000417 | Calycosin | 47.75 | 0.24 | 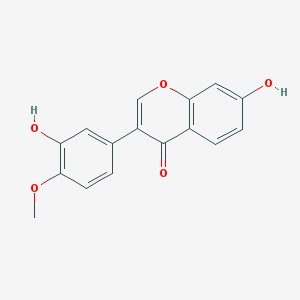 | GL |
| MOL004328 | naringenin | 59.29 | 0.21 | 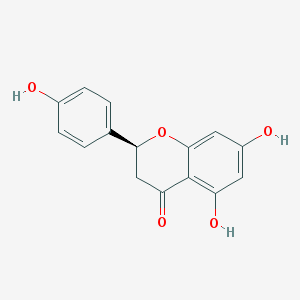 | GL |
| MOL004805 | (2S)-2-[4-hydroxy-3-(3-methylbut-2-enyl)phenyl]-8,8-dimethyl-2,3-dihydropyrano[2,3-f]chromen-4-one | 31.79 | 0.72 | 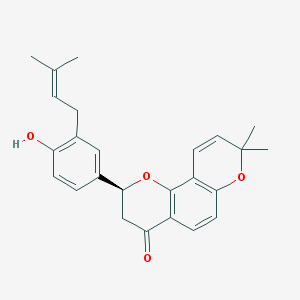 | GL |
| MOL004806 | euchrenone | 30.29 | 0.57 | 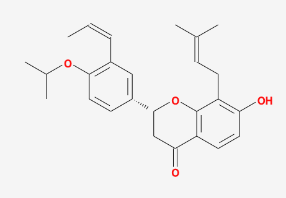 | GL |
| MOL004808 | glyasperin B | 65.22 | 0.44 | 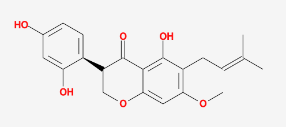 | GL |
| MOL004810 | glyasperin F | 75.84 | 0.54 | 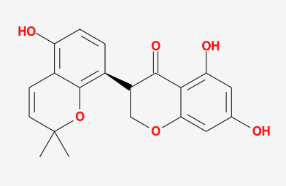 | GL |
| MOL004811 | Glyasperin C | 45.56 | 0.4 | 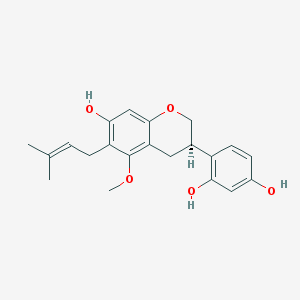 | GL |
| MOL004814 | Isotrifoliol | 31.94 | 0.42 | 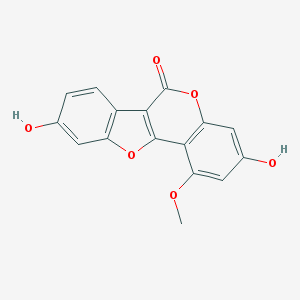 | GL |
| MOL004815 | (E)-1-(2,4-dihydroxyphenyl)-3-(2,2-dimethylchromen-6-yl)prop-2-en-1-one | 39.62 | 0.35 | 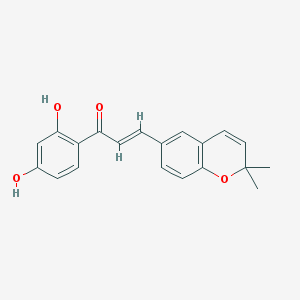 | GL |
| MOL004820 | kanzonols W | 50.48 | 0.52 | 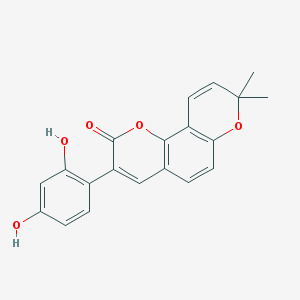 | GL |
| MOL004824 | (2S)-6-(2,4-dihydroxyphenyl)-2-(2-hydroxypropan-2-yl)-4-methoxy-2,3-dihydrofuro[3,2-g]chromen-7-one | 60.25 | 0.63 | 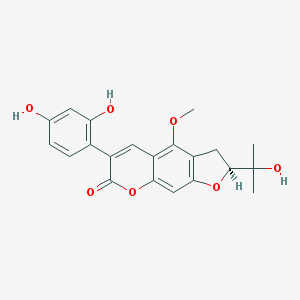 | GL |
| MOL004827 | Semilicoisoflavone B | 48.78 | 0.55 | 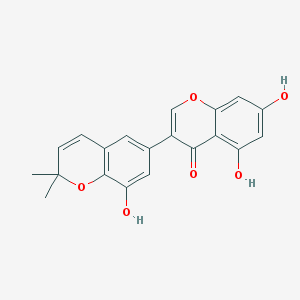 | GL |
| MOL004828 | Glepidotin A | 44.72 | 0.35 | 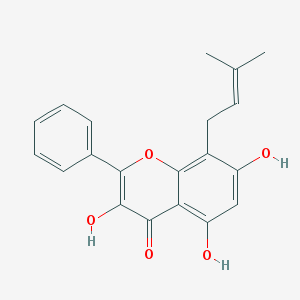 | GL |
| MOL004829 | Glepidotin B | 64.46 | 0.34 | 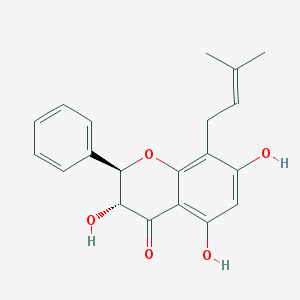 | GL |
| MOL004833 | Phaseolinisoflavan | 32.01 | 0.45 | 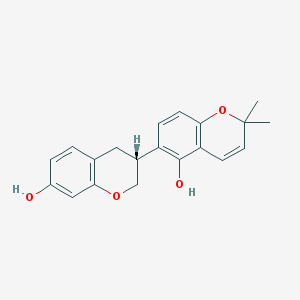 | GL |
| MOL004835 | Glypallichalcone | 61.6 | 0.19 | 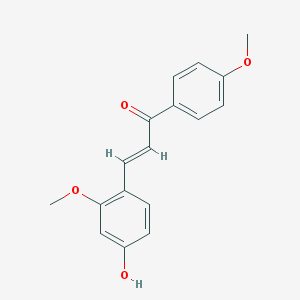 | GL |
| MOL004838 | 8-(6-hydroxy-2-benzofuranyl)-2,2-dimethyl-5-chromenol | 58.44 | 0.38 | 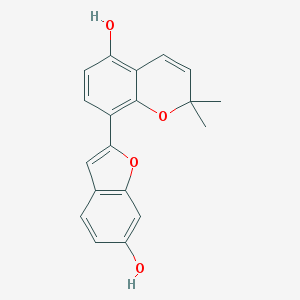 | GL |
| MOL004841 | Licochalcone B | 76.76 | 0.19 | 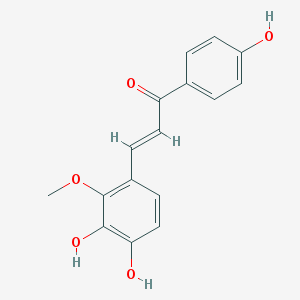 | GL |
| MOL004848 | licochalcone G | 49.25 | 0.32 |  | GL |
| MOL004849 | 3-(2,4-dihydroxyphenyl)-8-(1,1-dimethylprop-2-enyl)-7-hydroxy-5-methoxy-coumarin | 59.62 | 0.43 |  | GL |
| MOL004855 | Licoricone | 63.58 | 0.47 |  | GL |
| MOL004856 | Gancaonin A | 51.08 | 0.4 |  | GL |
| MOL004857 | Gancaonin B | 48.79 | 0.45 |  | GL |
| MOL004860 | licorice glycoside E | 32.89 | 0.27 |  | GL |
| MOL004863 | 3-(3,4-dihydroxyphenyl)-5,7-dihydroxy-8-(3-methylbut-2-enyl)chromone | 66.37 | 0.41 |  | GL |
| MOL004864 | 5,7-dihydroxy-3-(4-methoxyphenyl)-8-(3-methylbut-2-enyl)chromone | 30.49 | 0.41 |  | GL |
| MOL004866 | 2-(3,4-dihydroxyphenyl)-5,7-dihydroxy-6-(3-methylbut-2-enyl)chromone | 44.15 | 0.41 |  | GL |
| MOL004879 | Glycyrin | 52.61 | 0.47 |  | GL |
| MOL004882 | Licocoumarone | 33.21 | 0.36 |  | GL |
| MOL004883 | Licoisoflavone | 41.61 | 0.42 |  | GL |
| MOL004884 | Licoisoflavone B | 38.93 | 0.55 |  | GL |
| MOL004885 | licoisoflavanone | 52.47 | 0.54 |  | GL |
| MOL004891 | shinpterocarpin | 80.3 | 0.73 |  | GL |
| MOL004898 | (E)-3-[3,4-dihydroxy-5-(3-methylbut-2-enyl)phenyl]-1-(2,4-dihydroxyphenyl)prop-2-en-1-one | 46.27 | 0.31 |  | GL |
| MOL004903 | liquiritin | 65.69 | 0.74 |  | GL |
| MOL004904 | licopyranocoumarin | 80.36 | 0.65 |  | GL |
| MOL004905 | 3,22-Dihydroxy-11-oxo-delta(12)-oleanene-27-alpha-methoxycarbonyl-29-oic acid | 34.32 | 0.55 |  | GL |
| MOL004907 | Glyzaglabrin | 61.07 | 0.35 |  | GL |
| MOL004908 | Glabridin | 53.25 | 0.47 |  | GL |
| MOL004910 | Glabranin | 52.9 | 0.31 |  | GL |
| MOL004911 | Glabrene | 46.27 | 0.44 |  | GL |
| MOL004912 | Glabrone | 52.51 | 0.5 |  | GL |
| MOL004913 | 1,3-dihydroxy-9-methoxy-6-benzofurano[3,2-c]chromenone | 48.14 | 0.43 |  | GL |
| MOL004914 | 1,3-dihydroxy-8,9-dimethoxy-6-benzofurano[3,2-c]chromenone | 62.9 | 0.53 |  | GL |
| MOL004915 | Eurycarpin A | 43.28 | 0.37 |  | GL |
| MOL004917 | glycyroside | 37.25 | 0.79 |  | GL |
| MOL004924 | (-)-Medicocarpin | 40.99 | 0.95 |  | GL |
| MOL004935 | Sigmoidin-B | 34.88 | 0.41 |  | GL |
| MOL004941 | (2R)-7-hydroxy-2-(4-hydroxyphenyl)chroman-4-one | 71.12 | 0.18 |  | GL |
| MOL004945 | (2S)-7-hydroxy-2-(4-hydroxyphenyl)-8-(3-methylbut-2-enyl)chroman-4-one | 36.57 | 0.32 |  | GL |
| MOL004948 | Isoglycyrol | 44.7 | 0.84 |  | GL |
| MOL004949 | Isolicoflavonol | 45.17 | 0.42 |  | GL |
| MOL004957 | HMO | 38.37 | 0.21 |  | GL |
| MOL004959 | 1-Methoxyphaseollidin | 69.98 | 0.64 |  | GL |
| MOL004961 | Quercetin der. | 46.45 | 0.33 |  | GL |
| MOL004966 | 3'-Hydroxy-4'-O-Methylglabridin | 43.71 | 0.57 |  | GL |
| MOL000497 | licochalcone a | 40.79 | 0.29 |  | GL |
| MOL004974 | 3'-Methoxyglabridin | 46.16 | 0.57 |  | GL |
| MOL004978 | 2-[(3R)-8,8-dimethyl-3,4-dihydro-2H-pyrano[6,5-f]chromen-3-yl]-5-methoxyphenol | 36.21 | 0.52 |  | GL |
| MOL004980 | Inflacoumarin A | 39.71 | 0.33 |  | GL |
| MOL004985 | icos-5-enoic acid | 30.7 | 0.2 |  | GL |
| MOL004988 | Kanzonol F | 32.47 | 0.89 |  | GL |
| MOL004989 | 6-prenylated eriodictyol | 39.22 | 0.41 |  | GL |
| MOL004990 | 7,2',4'-trihydroxy－5-methoxy-3－arylcoumarin | 83.71 | 0.27 |  | GL |
| MOL004991 | 7-Acetoxy-2-methylisoflavone | 38.92 | 0.26 |  | GL |
| MOL004993 | 8-prenylated eriodictyol | 53.79 | 0.4 |  | GL |
| MOL004996 | gadelaidic acid | 30.7 | 0.2 |  | GL |
| MOL000500 | Vestitol | 74.66 | 0.21 |  | GL |
| MOL005000 | Gancaonin G | 60.44 | 0.39 |  | GL |
| MOL005001 | Gancaonin H | 50.1 | 0.78 |  | GL |
| MOL005003 | Licoagrocarpin | 58.81 | 0.58 |  | GL |
| MOL005007 | Glyasperins M | 72.67 | 0.59 |  | GL |
| MOL005008 | Glycyrrhiza flavonol A | 41.28 | 0.6 |  | GL |
| MOL005012 | Licoagroisoflavone | 57.28 | 0.49 |  | GL |
| MOL005013 | 18α-hydroxyglycyrrhetic acid | 41.16 | 0.71 |  | GL |
| MOL005016 | Odoratin | 49.95 | 0.3 |  | GL |
| MOL005017 | Phaseol | 78.77 | 0.58 |  | GL |
| MOL005018 | Xambioona | 54.85 | 0.87 |  | GL |
| MOL005020 | dehydroglyasperins C | 53.82 | 0.37 |  | GL |
| MOL000098 | quercetin | 46.43 | 0.28 |  | GL |

**Table S2.** Upregulated and downregulated genes in DEGs

| Upregulated genes | adj.P.Val | logFC |
| --- | --- | --- |
| GABRP | 8.04E-34 | 3.177064 |
| SLC6A14 | 1.26E-32 | 5.036665 |
| MMP7 | 2.85E-31 | 4.840438 |
| CHI3L1 | 2.98E-31 | 4.52905 |
| CXCR6 | 4.4E-31 | 1.279379 |
| PECAM1 | 2.28E-30 | 1.097425 |
| DUOX2 | 2.39E-30 | 4.098422 |
| PARP8 | 2.97E-30 | 1.465191 |
| LMAN1 | 9.22E-30 | 1.747889 |
| UBE2L6 | 1.07E-29 | 1.14 |
| C2 | 1.53E-29 | 1.693065 |
| CXCL1 | 2.23E-29 | 3.239323 |
| CD55 | 2.39E-29 | 1.628693 |
| HLA-DMA | 3.48E-29 | 1.200115 |
| LPCAT1 | 4.59E-29 | 1.473197 |
| DUOXA2 | 6.24E-29 | 4.184928 |
| TIMP1 | 6.99E-29 | 1.922829 |
| VNN1 | 9.62E-29 | 4.214382 |
| KYNU | 1.43E-28 | 2.608016 |
| OLFML2B | 2.16E-28 | 2.001578 |
| CEP112 | 4.87E-28 | 1.59049 |
| FEZ1 | 7.62E-28 | 1.8292 |
| CTSK | 8.52E-28 | 1.418005 |
| PSMB9 | 1.25E-27 | 1.025861 |
| PMEPA1 | 2.07E-27 | 1.158377 |
| PLA1A | 2.16E-27 | 2.345466 |
| GBP4 | 2.38E-27 | 1.638995 |
| IFNG | 2.55E-27 | 2.161213 |
| CXCL1 | 3.79E-27 | 2.812999 |
| CD44 | 3.81E-27 | 1.235131 |
| IFI44 | 6.66E-27 | 1.399831 |
| FSTL1 | 8.44E-27 | 1.069115 |
| RBPMS | 8.44E-27 | 1.473072 |
| DAPP1 | 8.5E-27 | 2.102443 |
| SAA1 | 9.94E-27 | 4.222814 |
| PDE4B | 1.17E-26 | 1.980138 |
| MGP | 1.61E-26 | 1.681287 |
| SELP | 1.62E-26 | 2.377924 |
| SAA1 | 1.74E-26 | 4.605455 |
| FKBP11 | 2.33E-26 | 1.34343 |
| IGHG1 | 2.39E-26 | 1.819643 |
| BGN | 2.74E-26 | 1.910916 |
| PCDH17 | 3.8E-26 | 2.221486 |
| IGHG3 | 4.1E-26 | 2.163073 |
| TGM2 | 4.48E-26 | 2.031049 |
| TRIM22 | 5.13E-26 | 1.473744 |
| ZFPM2 | 6E-26 | 1.692793 |
| TMEM158 | 6.55E-26 | 1.690826 |
| PIM2 | 6.58E-26 | 1.71802 |
| MMP10 | 6.98E-26 | 4.300694 |
| PRRX1 | 9.94E-26 | 2.974124 |
| IKBIP | 1.04E-25 | 1.451263 |
| FAP | 1.1E-25 | 2.721456 |
| ROBO1 | 1.12E-25 | 1.132206 |
| LST1 | 1.31E-25 | 1.041831 |
| CFB | 1.67E-25 | 1.813111 |
| GBP5 | 1.75E-25 | 2.209124 |
| ARHGEF3 | 1.78E-25 | 1.125181 |
| CHST3 | 2.02E-25 | 1.395368 |
| MMP3 | 2.24E-25 | 4.787171 |
| ROR2 | 2.36E-25 | 1.069094 |
| ELK3 | 2.76E-25 | 1.193848 |
| MTCL1 | 3.11E-25 | 1.786016 |
| SLFN11 | 3.11E-25 | 1.101099 |
| CXCL3 | 3.18E-25 | 2.588236 |
| SLC6A6 | 3.18E-25 | 1.201819 |
| MR1 | 3.31E-25 | 1.073987 |
| IDO1 | 3.53E-25 | 3.081441 |
| BACE2 | 3.8E-25 | 1.184828 |
| RGS5 | 4.01E-25 | 1.743159 |
| ASPHD2 | 4.01E-25 | 1.154301 |
| PDZK1IP1 | 4.27E-25 | 1.637714 |
| ARHGAP23 | 6.31E-25 | 1.335545 |
| OSBPL3 | 7.46E-25 | 1.273451 |
| CXCL8 | 7.77E-25 | 3.87441 |
| CLCN4 | 8.31E-25 | 1.517615 |
| XAF1 | 8.48E-25 | 1.012095 |
| LPIN1 | 1.1E-24 | 1.025008 |
| RPS6KA2 | 1.4E-24 | 1.121057 |
| SULF1 | 1.46E-24 | 1.343226 |
| IGFBP7 | 1.47E-24 | 1.527432 |
| ENTPD1 | 1.78E-24 | 1.088933 |
| C4BPB | 1.81E-24 | 2.158451 |
| KLHL5 | 2.52E-24 | 1.337351 |
| CXCL6 | 2.79E-24 | 3.732429 |
| RAB31 | 3.95E-24 | 1.129124 |
| XBP1 | 4.39E-24 | 1.15815 |
| VEGFC | 4.48E-24 | 1.453938 |
| RGCC | 4.76E-24 | 1.353738 |
| EDNRA | 5.48E-24 | 1.739904 |
| LYN | 6.35E-24 | 1.092461 |
| PLA2G4D | 6.35E-24 | 1.377006 |
| OAS2 | 6.88E-24 | 1.392692 |
| CTLA4 | 7.14E-24 | 2.748758 |
| ABCA12 | 8.51E-24 | 2.763819 |
| KDELR3 | 8.73E-24 | 1.489952 |
| SRD5A3 | 9.32E-24 | 1.20357 |
| CIITA | 9.76E-24 | 1.94878 |
| CALU | 9.81E-24 | 1.232787 |
| ZBP1 | 1.07E-23 | 2.353037 |
| ECSCR | 1.18E-23 | 1.400398 |
| MME | 1.38E-23 | 2.215393 |
| BAG2 | 1.4E-23 | 1.202202 |
| B9D1 | 1.42E-23 | 1.085815 |
| PHLDA1 | 1.47E-23 | 1.420579 |
| STAT1 | 1.52E-23 | 1.429294 |
| THY1 | 1.6E-23 | 1.858481 |
| APOL1 | 1.8E-23 | 1.419819 |
| IL33 | 1.82E-23 | 1.771913 |
| C1R | 1.96E-23 | 1.355023 |
| APOL3 | 2.01E-23 | 1.176993 |
| ANXA1 | 2.05E-23 | 1.274228 |
| HAPLN3 | 2.16E-23 | 2.320996 |
| NOD2 | 2.34E-23 | 1.594294 |
| GUCY1B3 | 2.49E-23 | 1.762665 |
| TRIB2 | 2.61E-23 | 1.644559 |
| S100A8 | 2.78E-23 | 3.268689 |
| DUSP4 | 2.82E-23 | 1.727724 |
| CREM | 3.27E-23 | 1.017344 |
| BCL2A1 | 3.56E-23 | 1.854979 |
| SLC6A20 | 3.87E-23 | 2.531085 |
| PI3 | 3.87E-23 | 2.511879 |
| PITPNC1 | 4.07E-23 | 1.346955 |
| FPR1 | 4.07E-23 | 2.735661 |
| TMEM45A | 4.37E-23 | 1.168853 |
| LRP8 | 4.43E-23 | 1.859367 |
| FAIM2 | 4.56E-23 | 1.626023 |
| MEOX1 | 4.69E-23 | 1.789129 |
| PEA15 | 5.06E-23 | 1.01448 |
| DYSF | 5.58E-23 | 1.574731 |
| CDH3 | 5.84E-23 | 3.444634 |
| MEOX2 | 6.59E-23 | 2.001845 |
| IGKV3-20 | 7.01E-23 | 1.820463 |
| ELL2 | 7.23E-23 | 1.149941 |
| TNIP3 | 7.62E-23 | 3.704103 |
| CXCL2 | 8E-23 | 2.519453 |
| FCGR3B | 8.29E-23 | 3.664859 |
| FILIP1L | 8.9E-23 | 1.151768 |
| RILPL2 | 9.61E-23 | 1.160012 |
| LRFN5 | 9.74E-23 | 1.347913 |
| SAMD9L | 1.01E-22 | 1.381639 |
| PDPN | 1.12E-22 | 2.298568 |
| ADAMTS6 | 1.38E-22 | 1.743252 |
| GBP1 | 1.46E-22 | 1.598153 |
| DRAM1 | 1.56E-22 | 1.182002 |
| NOS2 | 1.59E-22 | 2.771874 |
| THEMIS2 | 1.88E-22 | 1.303396 |
| BHLHE40 | 1.94E-22 | 1.203752 |
| SLC41A1 | 2.48E-22 | 1.165085 |
| IGKV3-11 | 2.52E-22 | 1.622186 |
| SLFN5 | 2.53E-22 | 1.067907 |
| SYT11 | 2.6E-22 | 1.526633 |
| COL6A3 | 2.65E-22 | 1.018028 |
| BHLHE22 | 2.73E-22 | 1.992546 |
| LCN2 | 2.83E-22 | 1.837586 |
| EBF3 | 2.96E-22 | 2.352812 |
| CXCL9 | 3.22E-22 | 3.057563 |
| FGR | 3.35E-22 | 2.11506 |
| SRGN | 3.46E-22 | 1.329702 |
| SAA2-SAA4 | 3.52E-22 | 4.134945 |
| DUOXA1 | 3.59E-22 | 2.021991 |
| AKAP2 | 4.09E-22 | 1.046072 |
| LCP2 | 4.18E-22 | 1.272784 |
| CFI | 4.2E-22 | 2.150675 |
| ST3GAL1 | 5.89E-22 | 1.368467 |
| ANGPT2 | 6.03E-22 | 1.3518 |
| CDH11 | 6.19E-22 | 1.282326 |
| GPR4 | 6.26E-22 | 1.881367 |
| COL4A1 | 6.3E-22 | 1.342993 |
| IGDCC4 | 6.49E-22 | 1.395931 |
| COL1A2 | 7.46E-22 | 1.269398 |
| TMEM263 | 7.46E-22 | 1.052867 |
| CSF2RB | 8.13E-22 | 1.176322 |
| IGKC | 8.29E-22 | 1.877314 |
| TMTC1 | 8.85E-22 | 1.11972 |
| TCN1 | 9.09E-22 | 3.324194 |
| KCNN3 | 9.74E-22 | 1.89468 |
| SGIP1 | 9.97E-22 | 1.696871 |
| PTPN13 | 9.99E-22 | 1.168831 |
| LAX1 | 1E-21 | 2.084294 |
| EML1 | 1.18E-21 | 1.262443 |
| GABBR1 | 1.2E-21 | 2.582863 |
| DEFB4A | 1.28E-21 | 4.826482 |
| FAM110B | 1.29E-21 | 1.123402 |
| SNCAIP | 1.74E-21 | 1.274344 |
| PLEK | 1.79E-21 | 1.442324 |
| PLAU | 1.79E-21 | 1.777092 |
| CCL4 | 1.93E-21 | 1.530024 |
| TMEM92 | 2.03E-21 | 1.429398 |
| ADGRE2 | 2.07E-21 | 1.826762 |
| STX11 | 2.12E-21 | 1.539421 |
| SMARCA1 | 2.26E-21 | 1.129909 |
| GPX8 | 2.42E-21 | 1.284965 |
| CST7 | 2.68E-21 | 1.332879 |
| GPX7 | 2.68E-21 | 1.555727 |
| SMCO2 | 2.74E-21 | 1.45631 |
| CHN1 | 2.76E-21 | 1.613519 |
| FBN1 | 2.84E-21 | 1.150529 |
| CD38 | 2.9E-21 | 1.897327 |
| TDO2 | 2.95E-21 | 1.953332 |
| NCF2 | 3.04E-21 | 1.235748 |
| GZMB | 3.06E-21 | 1.951928 |
| PDCD1LG2 | 3.1E-21 | 1.403938 |
| ADAMTS4 | 3.64E-21 | 2.606969 |
| TDRP | 3.66E-21 | 1.139504 |
| HEG1 | 3.91E-21 | 1.237148 |
| DUSP10 | 4.01E-21 | 1.15099 |
| DERL3 | 4.05E-21 | 1.693087 |
| CYSLTR1 | 4.1E-21 | 1.184284 |
| MEI1 | 4.11E-21 | 1.427472 |
| BTN2A2 | 4.2E-21 | 1.109616 |
| CTHRC1 | 4.29E-21 | 1.939923 |
| AQP3 | 4.61E-21 | 1.174935 |
| GNA15 | 4.72E-21 | 1.578652 |
| SLC2A14 | 4.78E-21 | 1.590315 |
| SMOX | 5.09E-21 | 1.019684 |
| CATSPERB | 5.85E-21 | 2.091893 |
| VCAM1 | 6.05E-21 | 1.036584 |
| ITGAX | 6.15E-21 | 1.671641 |
| RHOQ | 6.79E-21 | 1.044846 |
| ARNTL2 | 7.02E-21 | 1.688351 |
| KCNJ8 | 7.75E-21 | 1.689902 |
| TWIST1 | 8.35E-21 | 1.88897 |
| FUT8 | 9.34E-21 | 1.039294 |
| IL1RN | 9.71E-21 | 2.838027 |
| CCDC69 | 9.77E-21 | 1.154543 |
| FPR2 | 1.1E-20 | 3.335234 |
| CALCRL | 1.15E-20 | 1.544714 |
| NXPE3 | 1.19E-20 | 1.461334 |
| CPXM1 | 1.3E-20 | 1.649808 |
| EIF5A2 | 1.38E-20 | 1.38103 |
| WISP1 | 1.45E-20 | 2.454049 |
| PIK3R3 | 1.5E-20 | 1.054257 |
| STC1 | 1.51E-20 | 2.074943 |
| IRAK3 | 1.54E-20 | 1.306073 |
| GUCY1A3 | 1.58E-20 | 1.422448 |
| TNFSF13B | 1.69E-20 | 1.162225 |
| SNX10 | 1.75E-20 | 1.294246 |
| ICAM2 | 1.83E-20 | 1.120272 |
| ALPL | 1.89E-20 | 1.714369 |
| LIPG | 2.05E-20 | 1.027998 |
| RASGRP1 | 2.08E-20 | 1.395226 |
| ARHGAP29 | 2.19E-20 | 1.076427 |
| FCRL5 | 2.35E-20 | 1.898678 |
| GPR65 | 2.51E-20 | 1.221017 |
| DBN1 | 2.61E-20 | 1.05975 |
| ADGRL2 | 2.65E-20 | 1.352515 |
| FYN | 2.68E-20 | 1.225501 |
| CCL2 | 2.84E-20 | 1.456568 |
| SLPI | 3.11E-20 | 1.586223 |
| C2CD4B | 3.16E-20 | 1.811555 |
| SAA2 | 3.21E-20 | 3.702905 |
| CHRDL2 | 3.23E-20 | 2.397442 |
| IFI16 | 3.29E-20 | 1.066663 |
| SLAMF1 | 3.35E-20 | 1.350636 |
| AQP9 | 3.37E-20 | 3.379475 |
| SLC7A5 | 3.37E-20 | 1.471095 |
| ZNF333 | 4.17E-20 | 1.412277 |
| GPR176 | 4.31E-20 | 1.465111 |
| PDE10A | 4.57E-20 | 1.524221 |
| MMP9 | 4.81E-20 | 2.083022 |
| CPZ | 4.92E-20 | 1.267525 |
| PLA2G2A | 5.25E-20 | 1.922737 |
| ADPRH | 5.63E-20 | 1.082304 |
| DOC2B | 5.74E-20 | 1.602486 |
| PVRL4 | 5.9E-20 | 1.111475 |
| TPK1 | 5.98E-20 | 1.109745 |
| FCGR3A | 6.08E-20 | 2.481253 |
| ELOVL5 | 6.1E-20 | 1.053847 |
| PDGFRB | 6.34E-20 | 1.261595 |
| GLCCI1 | 6.65E-20 | 1.121525 |
| SGMS1 | 7.33E-20 | 1.182521 |
| SERPINB5 | 7.46E-20 | 3.445761 |
| RAB8B | 7.46E-20 | 1.111976 |
| CYYR1 | 7.49E-20 | 1.067417 |
| MZB1 | 7.58E-20 | 1.731738 |
| REG1A | 7.59E-20 | 4.394503 |
| C17orf107 | 7.62E-20 | 1.049126 |
| IQCG | 7.79E-20 | 1.056767 |
| REG3A | 8.59E-20 | 4.899805 |
| EGR3 | 9.05E-20 | 2.148426 |
| CSGALNACT1 | 1.03E-19 | 1.38478 |
| CXCR2 | 1.05E-19 | 2.03851 |
| CRNDE | 1.17E-19 | 1.377167 |
| ST3GAL5 | 1.21E-19 | 1.083815 |
| GFPT2 | 1.26E-19 | 1.741827 |
| CLEC4E | 1.29E-19 | 2.276165 |
| FAM155A | 1.32E-19 | 1.24712 |
| SERPINI1 | 1.33E-19 | 1.290556 |
| IGHM | 1.41E-19 | 2.612634 |
| FBXO16 | 1.42E-19 | 1.022799 |
| FOXP3 | 1.44E-19 | 1.553925 |
| IL1B | 1.52E-19 | 2.626654 |
| SERPING1 | 1.61E-19 | 1.259382 |
| CHST11 | 1.67E-19 | 1.088147 |
| RAMP3 | 1.68E-19 | 1.241504 |
| ODF3B | 1.84E-19 | 1.173204 |
| CLEC1A | 1.87E-19 | 1.030244 |
| S100P | 1.99E-19 | 1.61475 |
| FCGR3A | 2.03E-19 | 2.102728 |
| PCSK1 | 2.13E-19 | 2.561728 |
| TNFRSF9 | 2.24E-19 | 1.531424 |
| ITGA5 | 2.31E-19 | 1.458513 |
| GMFG | 2.55E-19 | 1.023964 |
| TRIM29 | 3.06E-19 | 2.614203 |
| CXCL11 | 3.16E-19 | 2.513238 |
| PRF1 | 3.26E-19 | 1.051923 |
| RAB36 | 3.29E-19 | 1.080452 |
| FFAR2 | 3.34E-19 | 1.975059 |
| PCOLCE | 3.45E-19 | 1.283829 |
| TFAP2C | 3.45E-19 | 1.598216 |
| RAC2 | 3.52E-19 | 1.193971 |
| DEFA6 | 3.58E-19 | 4.575058 |
| IGFBP5 | 3.66E-19 | 1.334653 |
| SACS | 3.76E-19 | 1.072747 |
| CXCR2 | 3.78E-19 | 2.532938 |
| SERPINE1 | 3.79E-19 | 2.154067 |
| NR4A3 | 3.84E-19 | 2.216241 |
| SAMSN1 | 4.17E-19 | 1.133799 |
| LDLRAD3 | 4.26E-19 | 1.18009 |
| GRHL1 | 4.49E-19 | 1.447936 |
| CD40 | 4.79E-19 | 1.440936 |
| CHST2 | 4.98E-19 | 1.759968 |
| ICOS | 5.15E-19 | 1.796394 |
| IL1A | 5.28E-19 | 2.242508 |
| NNMT | 5.29E-19 | 1.708599 |
| CD2 | 5.52E-19 | 1.2605 |
| RTEL1-TNFRSF6B | 5.65E-19 | 1.515133 |
| S100A9 | 5.66E-19 | 2.101816 |
| RASSF5 | 5.75E-19 | 1.252796 |
| PLS3 | 5.93E-19 | 1.021373 |
| STMN3 | 6.16E-19 | 1.243357 |
| TMEM200C | 6.64E-19 | 1.800162 |
| KLF7 | 7E-19 | 1.102041 |
| C2CD4A | 7.03E-19 | 2.681377 |
| FADS1 | 7.07E-19 | 1.595222 |
| ME1 | 7.28E-19 | 1.238877 |
| IGKV1-12 | 7.93E-19 | 1.476425 |
| MMRN1 | 8.12E-19 | 1.854074 |
| REG4 | 8.22E-19 | 1.878659 |
| NOTCH3 | 8.52E-19 | 1.342593 |
| THBD | 8.61E-19 | 1.252434 |
| WARS | 9.11E-19 | 1.366419 |
| IL26 | 9.65E-19 | 1.703893 |
| EBF1 | 9.93E-19 | 1.468219 |
| ADGRF5 | 9.93E-19 | 1.199176 |
| CADM1 | 1.19E-18 | 2.034873 |
| FCGR1A | 1.21E-18 | 1.7759 |
| GIMAP4 | 1.23E-18 | 1.143592 |
| CDH5 | 1.25E-18 | 1.293489 |
| TNC | 1.25E-18 | 2.267624 |
| ORAI2 | 1.29E-18 | 1.41929 |
| PPP1R18 | 1.35E-18 | 1.159805 |
| MAGEH1 | 1.42E-18 | 1.057079 |
| TCF4 | 1.42E-18 | 1.156187 |
| LSAMP | 1.46E-18 | 1.2663 |
| SELPLG | 1.49E-18 | 1.03627 |
| SOCS1 | 1.52E-18 | 1.743409 |
| LILRB2 | 1.54E-18 | 1.659734 |
| SPOCK2 | 1.57E-18 | 1.196186 |
| CD93 | 1.63E-18 | 1.294893 |
| DPYSL3 | 1.64E-18 | 1.34512 |
| ITGAM | 1.69E-18 | 1.160087 |
| CARD6 | 1.71E-18 | 1.515158 |
| STRA6 | 1.98E-18 | 1.668179 |
| CLDN2 | 2.03E-18 | 2.687711 |
| SLC9B2 | 2.06E-18 | 1.248531 |
| AMOTL1 | 2.13E-18 | 1.034179 |
| WIPF1 | 2.22E-18 | 1.142501 |
| ATP8B2 | 2.37E-18 | 1.17072 |
| ANGPTL2 | 2.43E-18 | 1.55677 |
| THSD7A | 2.53E-18 | 1.257933 |
| ZC3H12A | 2.55E-18 | 1.424896 |
| GJC1 | 2.58E-18 | 1.621004 |
| SLA | 2.72E-18 | 1.492869 |
| CCDC3 | 2.79E-18 | 1.816834 |
| MNDA | 2.85E-18 | 1.739313 |
| APOBEC3G | 2.98E-18 | 1.414411 |
| APCDD1 | 3.05E-18 | 1.124269 |
| TRPS1 | 3.18E-18 | 1.061157 |
| EDEM1 | 3.22E-18 | 1.035415 |
| ADAM19 | 3.27E-18 | 1.13802 |
| TAL1 | 3.32E-18 | 1.0626 |
| SOCS3 | 3.46E-18 | 2.089667 |
| PAPLN | 3.49E-18 | 1.120921 |
| SERPINA3 | 3.56E-18 | 2.804339 |
| PLEKHO2 | 3.57E-18 | 1.04592 |
| FAM124A | 3.64E-18 | 1.265929 |
| CH25H | 3.77E-18 | 1.570186 |
| OSMR | 3.8E-18 | 1.30342 |
| SERPINB9 | 4.17E-18 | 1.099578 |
| TTC9 | 4.21E-18 | 2.046173 |
| ADA | 4.31E-18 | 1.248262 |
| NTNG2 | 4.48E-18 | 1.147274 |
| EGR2 | 4.48E-18 | 1.845487 |
| S1PR1 | 4.63E-18 | 1.475783 |
| RGS4 | 5.02E-18 | 2.072619 |
| SIX4 | 5.61E-18 | 1.20428 |
| KLHL6 | 5.62E-18 | 1.2526 |
| CD82 | 5.65E-18 | 1.027278 |
| SLC7A11 | 5.69E-18 | 1.346101 |
| PRDM1 | 6.23E-18 | 1.42273 |
| PRKCH | 6.54E-18 | 1.042622 |
| G0S2 | 6.97E-18 | 1.363719 |
| ZG16B | 8.1E-18 | 1.236897 |
| BCL6B | 8.39E-18 | 1.087061 |
| NOX4 | 8.77E-18 | 1.254817 |
| LILRB1 | 9.02E-18 | 1.594742 |
| C17orf96 | 9.85E-18 | 1.24576 |
| STEAP4 | 1.01E-17 | 1.350478 |
| SH3BP5 | 1.04E-17 | 1.062253 |
| APLNR | 1.11E-17 | 1.195146 |
| ADAMTS1 | 1.18E-17 | 1.406484 |
| RORA | 1.19E-17 | 1.024956 |
| EMP3 | 1.28E-17 | 1.013331 |
| AJUBA | 1.31E-17 | 1.115764 |
| ABCC9 | 1.36E-17 | 1.655751 |
| AIM2 | 1.36E-17 | 1.805721 |
| CYP4X1 | 1.36E-17 | 1.331848 |
| LDB2 | 1.4E-17 | 1.004686 |
| TMEM154 | 1.46E-17 | 1.200878 |
| FAM49A | 1.62E-17 | 1.148128 |
| CYP7B1 | 1.66E-17 | 1.406637 |
| LRRC8C | 1.7E-17 | 1.168844 |
| COL5A2 | 1.84E-17 | 1.184678 |
| SLC17A9 | 1.93E-17 | 1.411457 |
| CXCL10 | 1.97E-17 | 2.493637 |
| PTPRC | 2.06E-17 | 1.230559 |
| JAK3 | 2.14E-17 | 1.739376 |
| LOC102724428 | 2.18E-17 | 1.781165 |
| ICAM1 | 2.2E-17 | 1.508282 |
| PLEKHS1 | 2.27E-17 | 1.314177 |
| VNN3 | 2.31E-17 | 2.022511 |
| LAMP3 | 2.42E-17 | 1.880446 |
| ERO1A | 2.44E-17 | 1.213106 |
| FAM83A | 2.48E-17 | 1.559679 |
| ADAMTS9 | 2.51E-17 | 1.15557 |
| F2RL2 | 2.58E-17 | 1.869963 |
| SP110 | 2.71E-17 | 1.082677 |
| ANTXR1 | 2.78E-17 | 1.351655 |
| PF4 | 2.92E-17 | 1.091881 |
| IL23A | 2.95E-17 | 1.081615 |
| ANKRD36 | 3E-17 | 1.28479 |
| LTBP2 | 3.14E-17 | 1.64254 |
| IGKV2-28 | 3.25E-17 | 1.672714 |
| C4A | 3.4E-17 | 1.593915 |
| FAM92B | 3.43E-17 | 1.94111 |
| GPR183 | 3.51E-17 | 1.460839 |
| CCR6 | 3.77E-17 | 1.858863 |
| SOD3 | 3.84E-17 | 1.144053 |
| OLFM1 | 3.84E-17 | 1.700675 |
| ESR1 | 3.89E-17 | 1.245349 |
| FAM126A | 3.91E-17 | 1.073227 |
| CACNA1C | 3.94E-17 | 1.296675 |
| PNOC | 4.01E-17 | 1.624665 |
| KDR | 4.02E-17 | 1.089141 |
| CMTM7 | 4.05E-17 | 1.164039 |
| SORD | 4.18E-17 | 1.038538 |
| SLC28A3 | 4.23E-17 | 1.187466 |
| TACSTD2 | 4.25E-17 | 1.872145 |
| CLDN11 | 4.53E-17 | 1.274233 |
| FCGR1B | 4.95E-17 | 1.348406 |
| MMP12 | 5.06E-17 | 1.584231 |
| SERPINA1 | 5.07E-17 | 2.393757 |
| SLC35G2 | 5.12E-17 | 1.177382 |
| ANKRD36BP2 | 5.16E-17 | 1.167579 |
| ADORA2A | 5.37E-17 | 1.085567 |
| SLC5A1 | 6.47E-17 | 1.089488 |
| PTGFR | 6.49E-17 | 1.236007 |
| ADCYAP1 | 6.56E-17 | 2.275292 |
| ADRBK2 | 6.81E-17 | 1.006619 |
| AGPAT4 | 7.56E-17 | 1.379915 |
| VNN2 | 7.74E-17 | 1.497612 |
| HCLS1 | 7.98E-17 | 1.164355 |
| LCP1 | 8.09E-17 | 1.112336 |
| GPR171 | 8.41E-17 | 1.006979 |
| SH2D1A | 8.46E-17 | 1.553302 |
| COL22A1 | 8.69E-17 | 1.562043 |
| IRAK2 | 8.97E-17 | 1.252022 |
| KLF2 | 9.09E-17 | 1.219638 |
| TFPI | 9.51E-17 | 1.055915 |
| IGK | 9.54E-17 | 1.231792 |
| ESM1 | 9.62E-17 | 1.498001 |
| TNFAIP8 | 9.73E-17 | 1.10828 |
| HTRA1 | 9.97E-17 | 1.000197 |
| SPINK4 | 1.1E-16 | 1.56636 |
| KCNE4 | 1.13E-16 | 1.161296 |
| IFNAR2 | 1.19E-16 | 1.126415 |
| SEMA4A | 1.2E-16 | 1.076926 |
| CD3D | 1.23E-16 | 1.263392 |
| CXCL17 | 1.24E-16 | 1.749258 |
| RASSF2 | 1.24E-16 | 1.119492 |
| EMCN | 1.25E-16 | 1.060041 |
| IGLJ3 | 1.39E-16 | 1.848069 |
| BASP1 | 1.5E-16 | 1.055893 |
| BATF3 | 1.52E-16 | 1.12319 |
| CHST15 | 1.53E-16 | 1.027041 |
| HECW2 | 1.54E-16 | 1.300473 |
| ITGB2 | 1.65E-16 | 1.116481 |
| CLCF1 | 1.66E-16 | 1.0636 |
| BNC2 | 1.7E-16 | 1.263945 |
| NLRP1 | 1.71E-16 | 1.292307 |
| ITPR1 | 1.81E-16 | 1.057226 |
| VGLL3 | 1.83E-16 | 1.257762 |
| EVI2B | 2.01E-16 | 1.15988 |
| NRIP2 | 2.04E-16 | 1.066804 |
| ELMO1 | 2.21E-16 | 1.009414 |
| FLRT2 | 2.31E-16 | 1.440556 |
| PPP1R16B | 2.31E-16 | 1.312434 |
| MAFF | 2.33E-16 | 1.126832 |
| ANXA6 | 2.34E-16 | 1.090632 |
| ST8SIA1 | 2.37E-16 | 1.24045 |
| HLA-DOA | 2.41E-16 | 1.240556 |
| MLLT11 | 2.48E-16 | 1.126492 |
| ADGRL4 | 2.48E-16 | 1.202785 |
| CD86 | 2.55E-16 | 1.006937 |
| PALMD | 2.64E-16 | 1.178244 |
| RFTN1 | 2.71E-16 | 1.185699 |
| COL1A1 | 2.85E-16 | 1.601894 |
| SP140 | 2.89E-16 | 1.738514 |
| IL12RB1 | 2.91E-16 | 1.118194 |
| ADAM12 | 2.92E-16 | 1.179028 |
| CSRP2 | 3.09E-16 | 1.314912 |
| RBP5 | 3.12E-16 | 1.085851 |
| IGHM | 3.17E-16 | 1.109244 |
| SPARC | 3.19E-16 | 1.052447 |
| PTGDS | 3.44E-16 | 2.06458 |
| SH2D2A | 3.47E-16 | 1.003053 |
| ARL4C | 3.55E-16 | 1.054935 |
| FMNL1 | 3.86E-16 | 1.380219 |
| ACKR4 | 4.23E-16 | 1.642591 |
| FXYD5 | 4.25E-16 | 1.029437 |
| RNF183 | 4.27E-16 | 1.476802 |
| DOCK8 | 4.33E-16 | 1.176744 |
| CETP | 4.43E-16 | 1.492322 |
| FGF7 | 4.44E-16 | 1.071462 |
| SLCO4A1 | 4.47E-16 | 1.273507 |
| TMPRSS3 | 4.81E-16 | 1.28206 |
| RAMP2 | 5.11E-16 | 1.273164 |
| LRRK2 | 5.12E-16 | 1.39459 |
| KCNA3 | 5.91E-16 | 1.279424 |
| FGFR1 | 6.01E-16 | 1.100658 |
| ENKUR | 6.28E-16 | 1.989241 |
| SLC16A4 | 6.3E-16 | 1.531946 |
| MCAM | 6.44E-16 | 1.168597 |
| SLCO5A1 | 6.84E-16 | 1.120335 |
| HLA-DPB1 | 6.96E-16 | 1.060865 |
| TOX2 | 7.12E-16 | 1.33183 |
| LAMC2 | 7.15E-16 | 1.006749 |
| TPTE2 | 7.19E-16 | 1.067148 |
| HHEX | 7.24E-16 | 1.154993 |
| LYPD5 | 7.3E-16 | 1.585176 |
| ACKR1 | 7.31E-16 | 1.02885 |
| C4BPA | 7.97E-16 | 2.625567 |
| DMBT1 | 8.24E-16 | 2.167561 |
| HSD11B1 | 8.48E-16 | 1.812739 |
| SEC14L2 | 8.62E-16 | 1.155739 |
| HLA-DOB | 9.27E-16 | 1.801371 |
| CFP | 9.66E-16 | 1.099058 |
| DEFA5 | 1.01E-15 | 4.079678 |
| EFEMP1 | 1.04E-15 | 1.037151 |
| HMSD | 1.04E-15 | 1.097176 |
| PTP4A3 | 1.1E-15 | 1.501101 |
| SLAMF7 | 1.1E-15 | 1.044024 |
| CD248 | 1.14E-15 | 1.097308 |
| FAM167A | 1.14E-15 | 1.422191 |
| IGLL5 | 1.16E-15 | 1.478897 |
| GLIPR1 | 1.3E-15 | 1.135748 |
| MMP19 | 1.35E-15 | 1.418334 |
| KMO | 1.36E-15 | 1.434507 |
| CD34 | 1.37E-15 | 1.070403 |
| IFIT3 | 1.44E-15 | 1.055781 |
| SELL | 1.44E-15 | 2.332916 |
| CYTIP | 1.45E-15 | 1.279645 |
| MXRA5 | 1.55E-15 | 1.060849 |
| NXN | 1.61E-15 | 1.076716 |
| VCAN | 1.71E-15 | 1.019061 |
| IL2RB | 1.75E-15 | 1.101274 |
| UNC5CL | 1.84E-15 | 1.030409 |
| TLL2 | 1.86E-15 | 1.135569 |
| PRSS2 | 1.91E-15 | 3.195317 |
| ASRGL1 | 2.05E-15 | 1.056805 |
| CDK14 | 2.06E-15 | 1.075819 |
| B3GALNT1 | 2.06E-15 | 1.183818 |
| LGI2 | 2.3E-15 | 2.054696 |
| PIK3CD | 2.37E-15 | 1.2072 |
| FAM20A | 2.4E-15 | 1.308284 |
| CXorf36 | 2.41E-15 | 1.03504 |
| LILRA6 | 2.47E-15 | 1.369683 |
| CD80 | 2.5E-15 | 1.592103 |
| SIRPB1 | 2.51E-15 | 1.268357 |
| EHD3 | 2.52E-15 | 1.148424 |
| ARID5A | 2.58E-15 | 1.116109 |
| GEM | 2.6E-15 | 1.353986 |
| CD1B | 2.62E-15 | 1.553416 |
| ZNF469 | 2.74E-15 | 1.062504 |
| RHOH | 2.74E-15 | 1.445287 |
| MOXD1 | 2.75E-15 | 1.146067 |
| CD27 | 2.93E-15 | 1.311756 |
| NEBL | 2.97E-15 | 1.046158 |
| TIGIT | 3.53E-15 | 1.227766 |
| F5 | 3.54E-15 | 1.271821 |
| EMB | 3.56E-15 | 1.205133 |
| MX2 | 3.61E-15 | 1.156589 |
| TMEM163 | 4.04E-15 | 2.241182 |
| CLDN14 | 4.13E-15 | 1.048163 |
| TLR8 | 4.15E-15 | 1.041051 |
| NFAM1 | 4.19E-15 | 1.028466 |
| TIMP3 | 4.42E-15 | 1.273897 |
| ARHGAP25 | 4.5E-15 | 1.062064 |
| C3 | 4.75E-15 | 2.135828 |
| IFI44L | 4.77E-15 | 1.259459 |
| VSIG1 | 5.1E-15 | 1.937856 |
| STRIP2 | 5.14E-15 | 1.430374 |
| SLC1A3 | 5.26E-15 | 1.302073 |
| RAB39B | 5.32E-15 | 1.512388 |
| CBR3 | 5.45E-15 | 1.091876 |
| FIBIN | 5.72E-15 | 1.733803 |
| NLRP7 | 5.84E-15 | 1.546372 |
| IL21R | 6.38E-15 | 1.690789 |
| TMEM71 | 6.46E-15 | 1.157819 |
| OLFM4 | 6.49E-15 | 1.647672 |
| TESC | 6.83E-15 | 1.135037 |
| TREM1 | 7.27E-15 | 1.736061 |
| STS | 7.55E-15 | 1.197634 |
| SPAG4 | 7.61E-15 | 1.518194 |
| P2RY8 | 7.8E-15 | 1.253795 |
| ST8SIA4 | 8.03E-15 | 1.001734 |
| TRBC1 | 8.52E-15 | 1.217587 |
| LILRB4 | 8.79E-15 | 1.316 |
| BMP7 | 8.91E-15 | 1.30989 |
| LOXL2 | 9.01E-15 | 1.085692 |
| TRBC2 | 9.18E-15 | 1.149303 |
| TUBB6 | 9.35E-15 | 1.132238 |
| LOC100129518 | 9.7E-15 | 1.117417 |
| CYR61 | 9.85E-15 | 2.372353 |
| TAGAP | 1.07E-14 | 1.095115 |
| SELE | 1.08E-14 | 2.328468 |
| PROK2 | 1.08E-14 | 2.682521 |
| MICB | 1.18E-14 | 1.040654 |
| LIMD2 | 1.23E-14 | 1.546642 |
| IL6 | 1.27E-14 | 2.329172 |
| EOMES | 1.27E-14 | 1.509195 |
| CASP1 | 1.29E-14 | 1.025482 |
| SNPH | 1.37E-14 | 1.035384 |
| KRT6B | 1.39E-14 | 2.333316 |
| COL12A1 | 1.45E-14 | 1.432642 |
| P2RY13 | 1.45E-14 | 1.121303 |
| KIAA0125 | 1.5E-14 | 1.513121 |
| SH3TC2 | 1.52E-14 | 1.169666 |
| CHI3L2 | 1.54E-14 | 1.975606 |
| TSHZ2 | 1.61E-14 | 1.149321 |
| AGT | 1.62E-14 | 1.417366 |
| SLAMF6 | 1.73E-14 | 1.341866 |
| ECM2 | 1.82E-14 | 1.099886 |
| EMB | 1.85E-14 | 1.076031 |
| VWA1 | 1.94E-14 | 1.045869 |
| GJA4 | 1.95E-14 | 1.289477 |
| ARHGAP30 | 1.96E-14 | 1.273434 |
| IL2RA | 2.07E-14 | 1.668662 |
| TRAC | 2.11E-14 | 1.177764 |
| LILRA1 | 2.14E-14 | 1.040811 |
| VWF | 2.17E-14 | 1.360623 |
| ISLR | 2.17E-14 | 1.131108 |
| PRR16 | 2.19E-14 | 1.354807 |
| FCN1 | 2.23E-14 | 1.415436 |
| AIF1L | 2.26E-14 | 1.075116 |
| KIAA0895 | 2.39E-14 | 1.081828 |
| PRSS1 | 2.41E-14 | 2.944016 |
| LILRB1 | 2.52E-14 | 1.255414 |
| PSAT1 | 2.59E-14 | 1.140033 |
| PTPN7 | 2.82E-14 | 1.195131 |
| IGLV1-41 | 2.83E-14 | 1.315204 |
| LPL | 2.96E-14 | 1.530732 |
| PLN | 2.98E-14 | 1.083181 |
| GZMK | 3.11E-14 | 1.647678 |
| ALPK2 | 3.15E-14 | 1.690493 |
| HCAR2 | 3.33E-14 | 2.356491 |
| SYTL1 | 3.54E-14 | 1.079757 |
| RGS16 | 4.01E-14 | 1.198723 |
| SASH3 | 4.03E-14 | 1.268063 |
| RAPGEF4 | 4.04E-14 | 1.261894 |
| YME1L1 | 4.22E-14 | 1.110559 |
| CSTA | 4.55E-14 | 1.189321 |
| KCNA2 | 4.57E-14 | 1.285455 |
| LBH | 4.57E-14 | 1.143283 |
| DUOX1 | 4.63E-14 | 1.23775 |
| MRAS | 4.67E-14 | 1.001412 |
| CD79A | 4.71E-14 | 1.924477 |
| RELT | 4.77E-14 | 1.093097 |
| CD53 | 4.94E-14 | 1.14773 |
| ADGRE1 | 5.03E-14 | 1.045091 |
| ITLN2 | 5.35E-14 | 3.257352 |
| PSTPIP1 | 5.42E-14 | 1.023436 |
| LYZ | 5.52E-14 | 1.324614 |
| PTGS2 | 5.63E-14 | 2.018696 |
| RASIP1 | 5.7E-14 | 1.044363 |
| IL13RA2 | 5.94E-14 | 2.234203 |
| GJA5 | 6.24E-14 | 1.342788 |
| FMNL3 | 6.38E-14 | 1.132325 |
| CREB5 | 6.41E-14 | 1.55386 |
| ACPP | 6.6E-14 | 1.293125 |
| SLAIN1 | 6.61E-14 | 1.217579 |
| CXCL5 | 6.85E-14 | 3.75453 |
| ADGRG6 | 6.99E-14 | 1.255068 |
| CCL18 | 7.32E-14 | 2.151955 |
| THBS2 | 7.65E-14 | 1.872967 |
| ITK | 7.76E-14 | 1.364339 |
| PRDM8 | 7.91E-14 | 1.06137 |
| PARVG | 8.29E-14 | 1.108783 |
| DMKN | 8.51E-14 | 1.188909 |
| CD5 | 9.14E-14 | 1.277738 |
| CCDC80 | 9.4E-14 | 1.126435 |
| RUNX3 | 9.45E-14 | 1.144287 |
| PPM1K | 9.98E-14 | 1.04625 |
| FER1L4 | 1.01E-13 | 1.60013 |
| IKZF1 | 1.02E-13 | 1.171251 |
| CCL3 | 1.03E-13 | 1.37502 |
| TWIST2 | 1.06E-13 | 1.032646 |
| APIP | 1.08E-13 | 1.119943 |
| SDR16C5 | 1.09E-13 | 1.342328 |
| ITGAL | 1.09E-13 | 1.15242 |
| AFAP1L1 | 1.12E-13 | 1.028408 |
| FKBP10 | 1.17E-13 | 1.036157 |
| SORCS2 | 1.18E-13 | 1.101073 |
| S100B | 1.18E-13 | 1.126984 |
| LHX6 | 1.22E-13 | 1.283552 |
| WAS | 1.22E-13 | 1.157966 |
| CLEC5A | 1.25E-13 | 1.458001 |
| CR1 | 1.29E-13 | 1.677328 |
| NCS1 | 1.35E-13 | 1.105276 |
| SIRPG | 1.38E-13 | 1.522036 |
| PILRA | 1.42E-13 | 1.008286 |
| FGF2 | 1.42E-13 | 1.008772 |
| TSPAN11 | 1.43E-13 | 1.122286 |
| LAIR2 | 1.58E-13 | 1.995848 |
| PLXDC1 | 1.66E-13 | 1.259065 |
| SLC26A4 | 1.67E-13 | 1.85671 |
| COL18A1 | 1.68E-13 | 1.201482 |
| MMP1 | 1.69E-13 | 1.968965 |
| CALD1 | 1.7E-13 | 1.247656 |
| GPSM3 | 1.71E-13 | 1.073007 |
| ARHGAP9 | 1.88E-13 | 1.165317 |
| S100A7 | 1.9E-13 | 2.653042 |
| RERG | 1.92E-13 | 1.122274 |
| CD52 | 2.05E-13 | 1.144248 |
| CORO1A | 2.27E-13 | 1.173253 |
| TLR2 | 2.27E-13 | 1.111491 |
| TNFRSF10C | 2.7E-13 | 1.249391 |
| FAM65B | 2.91E-13 | 1.727737 |
| ERMN | 3E-13 | 1.066973 |
| ADGRG3 | 3.21E-13 | 1.296584 |
| PLEKHO1 | 3.44E-13 | 1.052293 |
| KIFC3 | 3.6E-13 | 1.118071 |
| ETV7 | 3.62E-13 | 1.364319 |
| SKAP1 | 3.64E-13 | 1.068344 |
| IL7R | 4.03E-13 | 1.329657 |
| TPRG1 | 4.43E-13 | 1.314451 |
| CLDN1 | 4.53E-13 | 1.529227 |
| PKHD1L1 | 4.81E-13 | 1.126535 |
| ADM | 4.93E-13 | 1.046785 |
| TBXA2R | 4.94E-13 | 1.067089 |
| CCL3L1 | 5.13E-13 | 1.373952 |
| PRKCDBP | 5.31E-13 | 1.047393 |
| REG1B | 5.99E-13 | 4.147405 |
| ERP27 | 6.03E-13 | 1.253447 |
| SH2D3C | 6.05E-13 | 1.039599 |
| APBA2 | 6.68E-13 | 1.063924 |
| LEF1 | 7.08E-13 | 1.085074 |
| HBD | 7.33E-13 | 1.217641 |
| ZBED2 | 8.43E-13 | 1.513133 |
| TENM4 | 8.49E-13 | 1.122876 |
| LAMC1 | 8.69E-13 | 1.049468 |
| CLEC12A | 8.85E-13 | 1.061802 |
| ANK1 | 9.57E-13 | 1.03429 |
| ARSJ | 9.58E-13 | 1.029498 |
| CTSE | 9.69E-13 | 1.420659 |
| PHGDH | 1.01E-12 | 1.089341 |
| DOK3 | 1.02E-12 | 1.332657 |
| LOC728392 | 1.28E-12 | 1.061376 |
| GNLY | 1.34E-12 | 1.243946 |
| PTRF | 1.35E-12 | 1.021136 |
| COL4A4 | 1.38E-12 | 1.33862 |
| FAM26F | 1.42E-12 | 1.273275 |
| CLDN18 | 1.43E-12 | 1.702826 |
| S100A3 | 1.43E-12 | 1.122687 |
| TNFSF9 | 1.43E-12 | 1.041993 |
| PLA2G2D | 1.44E-12 | 2.119721 |
| IL17F | 1.51E-12 | 1.438358 |
| GREM1 | 1.52E-12 | 2.430697 |
| BTK | 1.71E-12 | 1.17002 |
| NMNAT2 | 1.72E-12 | 1.117177 |
| RGS1 | 1.8E-12 | 1.396278 |
| LYPD6B | 1.85E-12 | 1.261011 |
| FAM129A | 1.86E-12 | 1.123282 |
| PDLIM7 | 1.91E-12 | 1.098617 |
| KRT7 | 1.94E-12 | 1.260934 |
| CSF3R | 1.97E-12 | 1.605076 |
| FSCN1 | 1.98E-12 | 1.132544 |
| FCN3 | 2.03E-12 | 1.270745 |
| CYTH4 | 2.19E-12 | 1.023408 |
| ADGRG5 | 2.28E-12 | 1.096316 |
| KCNJ10 | 2.57E-12 | 1.325848 |
| TRAF3IP3 | 2.68E-12 | 1.143767 |
| CR1 | 2.92E-12 | 2.094308 |
| SPNS2 | 2.97E-12 | 1.3623 |
| CLEC4D | 3.04E-12 | 1.399412 |
| CCL11 | 3.3E-12 | 1.599747 |
| HVCN1 | 3.32E-12 | 1.317981 |
| CXCR1 | 3.45E-12 | 1.571818 |
| IL24 | 3.6E-12 | 2.070285 |
| TRBV9 | 3.63E-12 | 1.237157 |
| CD72 | 3.65E-12 | 1.934031 |
| SEMG1 | 3.7E-12 | 1.52294 |
| TNF | 3.87E-12 | 1.163945 |
| RND1 | 4.13E-12 | 1.210179 |
| COL14A1 | 5.21E-12 | 1.003266 |
| GAS1 | 5.37E-12 | 1.672681 |
| RASL11B | 5.53E-12 | 1.207966 |
| CD48 | 5.69E-12 | 1.016647 |
| CD3E | 5.83E-12 | 1.094919 |
| TFF1 | 5.98E-12 | 1.2627 |
| CD180 | 6.33E-12 | 1.203346 |
| LCK | 7.3E-12 | 1.05612 |
| SNAP25 | 7.32E-12 | 1.096909 |
| XKR9 | 7.6E-12 | 1.397834 |
| GPR84 | 7.76E-12 | 1.242752 |
| P2RY10 | 8.86E-12 | 1.452092 |
| CD3G | 8.92E-12 | 1.056978 |
| NFATC1 | 1.02E-11 | 1.408627 |
| TMEM156 | 1.06E-11 | 1.028757 |
| SLC16A14 | 1.28E-11 | 1.012877 |
| TRAF1 | 1.35E-11 | 1.084915 |
| KIAA0922 | 1.41E-11 | 1.165384 |
| DAZL | 1.43E-11 | 1.156393 |
| NLRC3 | 1.48E-11 | 1.015959 |
| PYHIN1 | 1.5E-11 | 1.152863 |
| LTB | 1.58E-11 | 1.512887 |
| RSPO3 | 1.86E-11 | 1.789126 |
| MAP4K1 | 1.96E-11 | 1.067441 |
| IL16 | 2.01E-11 | 1.144867 |
| CELSR1 | 2.19E-11 | 1.047912 |
| CD28 | 2.22E-11 | 1.268463 |
| SERPINB7 | 2.46E-11 | 3.184352 |
| TRAT1 | 2.49E-11 | 1.249917 |
| TRAV12-3 | 2.61E-11 | 1.446285 |
| CLCA3P | 2.62E-11 | 1.581276 |
| KLK12 | 2.88E-11 | 1.428748 |
| CXCR4 | 3.15E-11 | 1.338729 |
| S100A7 | 3.31E-11 | 2.024092 |
| CDKN2A | 3.34E-11 | 1.288885 |
| ALDH1A2 | 3.35E-11 | 2.277419 |
| IL17A | 4.05E-11 | 1.060729 |
| IGF2BP3 | 4.2E-11 | 1.441552 |
| PPP4R4 | 4.26E-11 | 1.262182 |
| APBB1IP | 4.4E-11 | 1.174981 |
| RNF43 | 4.59E-11 | 1.252948 |
| GALNT14 | 4.78E-11 | 1.215042 |
| FCMR | 4.8E-11 | 1.946369 |
| CD83 | 4.98E-11 | 1.369431 |
| FYB | 5.04E-11 | 1.25451 |
| CBLN3 | 5.76E-11 | 1.201238 |
| BTLA | 5.78E-11 | 1.229746 |
| GZMH | 5.84E-11 | 1.116109 |
| DNM1 | 5.98E-11 | 1.146329 |
| CCL22 | 6.21E-11 | 1.861203 |
| CMTM2 | 6.31E-11 | 1.406641 |
| P2RX5 | 6.75E-11 | 1.740626 |
| WDFY4 | 7.2E-11 | 1.284024 |
| PCED1B | 7.27E-11 | 1.212905 |
| FAM25BP | 7.55E-11 | 1.555856 |
| MEDAG | 7.73E-11 | 1.207661 |
| ACAP1 | 9.31E-11 | 1.191988 |
| SLCO1B3 | 9.69E-11 | 1.960765 |
| HGF | 9.82E-11 | 1.093183 |
| LTF | 1.05E-10 | 1.926546 |
| SLC2A6 | 1.19E-10 | 1.072567 |
| SUSD3 | 1.26E-10 | 1.064747 |
| IDO2 | 1.35E-10 | 1.106923 |
| IGLV8-61 | 1.42E-10 | 1.498862 |
| OSM | 1.54E-10 | 1.138045 |
| SIT1 | 1.62E-10 | 1.10264 |
| DCN | 1.65E-10 | 1.005204 |
| PARP15 | 1.7E-10 | 1.462684 |
| GATA3 | 1.84E-10 | 1.033528 |
| CEMIP | 1.86E-10 | 1.641804 |
| CD1A | 1.9E-10 | 1.521706 |
| ART3 | 2.1E-10 | 1.392032 |
| KCNJ15 | 2.17E-10 | 1.808565 |
| RASAL3 | 2.28E-10 | 1.089444 |
| LILRA2 | 2.75E-10 | 1.236304 |
| TUSC3 | 2.79E-10 | 1.009527 |
| HS6ST2 | 2.91E-10 | 1.026292 |
| EPHA3 | 3.15E-10 | 1.163709 |
| GAPT | 3.31E-10 | 1.09442 |
| PRKCB | 3.31E-10 | 1.038803 |
| CD37 | 3.48E-10 | 1.417325 |
| KLK11 | 3.53E-10 | 1.430026 |
| TNFSF11 | 3.7E-10 | 1.807994 |
| NDP | 3.7E-10 | 1.092159 |
| KRT17 | 3.94E-10 | 1.52623 |
| MADCAM1 | 4.09E-10 | 1.421468 |
| TLR10 | 4.19E-10 | 1.678426 |
| CCL4L1 | 4.26E-10 | 1.202842 |
| CRTAM | 4.59E-10 | 1.102956 |
| DTX1 | 4.83E-10 | 1.181744 |
| SYNPO2 | 5.64E-10 | 1.029772 |
| SPP1 | 5.94E-10 | 1.626806 |
| TESPA1 | 5.96E-10 | 1.233192 |
| BCAT1 | 6.61E-10 | 1.008641 |
| CD69 | 6.65E-10 | 1.003925 |
| CD1E | 6.82E-10 | 1.291275 |
| ASPN | 7.49E-10 | 1.146288 |
| POU2F2 | 8.49E-10 | 1.243037 |
| MFAP5 | 1.01E-09 | 1.572897 |
| SYNPR | 1.05E-09 | 1.181676 |
| NRG1 | 1.05E-09 | 1.345233 |
| GLT1D1 | 1.09E-09 | 1.186067 |
| TBC1D10C | 1.16E-09 | 1.236283 |
| IRF4 | 1.21E-09 | 1.157485 |
| C11orf21 | 1.24E-09 | 1.176319 |
| ROBO4 | 1.46E-09 | 1.01909 |
| CD300E | 1.51E-09 | 1.027168 |
| KCNMB1 | 1.51E-09 | 1.021373 |
| BANK1 | 1.58E-09 | 1.929508 |
| CD79B | 1.62E-09 | 1.461502 |
| S100A12 | 1.65E-09 | 2.31314 |
| ATF3 | 1.88E-09 | 1.184404 |
| FCRL3 | 2.17E-09 | 1.888684 |
| PLA2G3 | 2.19E-09 | 1.175785 |
| PCDH7 | 2.29E-09 | 1.120874 |
| TTN | 2.67E-09 | 1.276271 |
| TNFAIP6 | 2.84E-09 | 1.038528 |
| CD19 | 3.37E-09 | 1.838677 |
| CD84 | 3.72E-09 | 1.013803 |
| LTA | 3.88E-09 | 1.021705 |
| LY9 | 3.96E-09 | 1.099767 |
| GAL | 4.11E-09 | 1.485752 |
| FAM177B | 4.84E-09 | 1.114694 |
| PSMA8 | 5.14E-09 | 1.088902 |
| NCOA7 | 5.59E-09 | 1.089582 |
| ADGRF1 | 6.45E-09 | 1.56997 |
| RNASE2 | 6.66E-09 | 1.234081 |
| NMUR2 | 6.7E-09 | 1.936312 |
| NR4A1 | 8.85E-09 | 1.223712 |
| COL7A1 | 9.46E-09 | 1.241784 |
| ST3GAL4 | 1.18E-08 | 1.366448 |
| NR4A2 | 1.2E-08 | 1.200653 |
| FN1 | 1.24E-08 | 1.032179 |
| HDC | 1.33E-08 | 1.065845 |
| S100A7A | 2.02E-08 | 1.254492 |
| EGR1 | 2.14E-08 | 1.319049 |
| DSG3 | 2.2E-08 | 1.159311 |
| ANKRD55 | 2.36E-08 | 1.086362 |
| TMC8 | 2.49E-08 | 1.140186 |
| OLR1 | 3.4E-08 | 1.143709 |
| FCGR2B | 3.47E-08 | 1.097686 |
| FAM129C | 4.69E-08 | 2.096343 |
| FDCSP | 5.04E-08 | 2.650921 |
| CD1C | 5.15E-08 | 1.135531 |
| CCR7 | 6.71E-08 | 1.637605 |
| AHNAK2 | 7.12E-08 | 1.067137 |
| RASGRP2 | 7.45E-08 | 1.283639 |
| IGLV3-10 | 7.58E-08 | 1.57319 |
| FOS | 8.55E-08 | 1.04969 |
| SPINK5 | 8.57E-08 | 1.136847 |
| NRCAM | 1.08E-07 | 1.262684 |
| CCR3 | 1.09E-07 | 1.143433 |
| IKZF3 | 1.11E-07 | 1.224392 |
| STAP1 | 1.19E-07 | 1.281511 |
| SIGLEC5 | 1.73E-07 | 1.020869 |
| TFF2 | 1.8E-07 | 1.263506 |
| FOLH1 | 2.08E-07 | 1.339883 |
| PRTG | 2.16E-07 | 1.022617 |
| TPO | 2.55E-07 | 1.15728 |
| MS4A1 | 2.8E-07 | 2.172146 |
| ADGRG2 | 3.05E-07 | 1.113313 |
| CR2 | 3.66E-07 | 1.784012 |
| MASP1 | 4.89E-07 | 1.267704 |
| CXCL13 | 5.2E-07 | 2.199128 |
| IGHV3-13 | 5.82E-07 | 1.125289 |
| CCL20 | 6.63E-07 | 1.225223 |
| SERPINB3 | 6.99E-07 | 1.704925 |
| FCRL2 | 7.18E-07 | 1.041098 |
| BCAS4 | 7.31E-07 | 1.058206 |
| KRT6A | 7.93E-07 | 1.551696 |
| CD22 | 1.01E-06 | 1.574158 |
| SPRR1B | 1.04E-06 | 1.616009 |
| PLLP | 1.06E-06 | 1.022942 |
| OR51E2 | 1.12E-06 | 1.100076 |
| SFTPA2 | 1.13E-06 | 1.325788 |
| IL22RA2 | 1.13E-06 | 1.055786 |
| FCRL1 | 1.33E-06 | 1.517887 |
| C1orf228 | 1.5E-06 | 1.066793 |
| TFPI2 | 1.64E-06 | 1.170824 |
| TCL1A | 1.69E-06 | 1.822891 |
| FOXQ1 | 1.73E-06 | 1.350329 |
| CCL19 | 1.79E-06 | 1.801312 |
| MYEOV | 2.4E-06 | 1.263334 |
| SERPINB3 | 2.42E-06 | 1.5691 |
| SULT1C2 | 3.01E-06 | 1.429049 |
| KLK10 | 3.6E-06 | 1.904365 |
| SNX22 | 3.87E-06 | 1.176817 |
| BACH2 | 3.98E-06 | 1.295905 |
| HOXD13 | 4.28E-06 | 2.312193 |
| CXCR5 | 6.31E-06 | 1.246175 |
| MUC17 | 1.08E-05 | 1.123742 |
| PDX1 | 1.11E-05 | 1.088658 |
| PRAC1 | 1.27E-05 | 2.737324 |
| HOXB13 | 1.48E-05 | 2.057803 |
| KLK6 | 1.99E-05 | 1.318592 |
| VPREB3 | 0.000027 | 1.352383 |
| FOSB | 3.25E-05 | 1.532827 |
| FCRLA | 3.83E-05 | 1.275963 |
| GALNTL6 | 4.55E-05 | 1.265659 |
| ALDOB | 5.46E-05 | 1.453445 |
| TRIM40 | 0.000081 | 1.19431 |
| PRELP | 8.91E-05 | 1.111377 |
| CCL21 | 0.000364 | 1.177475 |
| IRX2 | 0.000437 | 1.013648 |
| NPSR1 | 0.0011 | 1.067382 |
| VIP | 0.00183 | 1.07917 |
| SLC28A2 | 0.002 | 1.143756 |
| GLDN | 0.00828 | 1.113522 |

| Downregulated genes | adj.P.Val | logFC |
| --- | --- | --- |
| ABCG2 | 8.04E-34 | -3.45406 |
| CCNJL | 8.45E-30 | -1.97325 |
| SLC23A1 | 8.92E-30 | -2.52775 |
| AQP7 | 9.22E-30 | -1.67883 |
| LRRN2 | 9.5E-30 | -2.12097 |
| AQP7 | 2.78E-29 | -3.15935 |
| SLC25A33 | 3.48E-29 | -1.01228 |
| TMEM63C | 4.49E-29 | -1.52677 |
| MS4A10 | 4.54E-29 | -3.48755 |
| PDXP | 4.59E-29 | -1.16296 |
| MEP1B | 7.05E-28 | -3.22202 |
| OSBPL1A | 9.09E-28 | -1.33565 |
| SLC25A34 | 1.1E-27 | -1.8837 |
| SLC35G1 | 1.1E-27 | -1.91509 |
| GCNT2 | 1.63E-27 | -1.65793 |
| DNAJB3 | 1.63E-27 | -2.27317 |
| METTL7B | 2E-27 | -1.94729 |
| SLC23A3 | 2.55E-27 | -2.02016 |
| ANKH | 3.06E-27 | -1.12048 |
| PLCD3 | 4.05E-27 | -1.00157 |
| FAM213A | 4.42E-27 | -1.40041 |
| LPCAT3 | 4.54E-27 | -1.05233 |
| EPB41L1 | 4.72E-27 | -1.26975 |
| ETFDH | 5.16E-27 | -1.05958 |
| PADI2 | 5.98E-27 | -1.81303 |
| ZSWIM3 | 6.39E-27 | -1.03001 |
| ZNF575 | 8.44E-27 | -2.01852 |
| OTOP2 | 8.44E-27 | -2.46111 |
| TNNC2 | 1.39E-26 | -1.20144 |
| MGAT4B | 1.39E-26 | -1.16939 |
| PRKG2 | 1.62E-26 | -2.04016 |
| ACOX1 | 2.39E-26 | -1.3122 |
| SGK2 | 2.43E-26 | -2.07839 |
| DNAJB3 | 2.73E-26 | -2.15936 |
| KCNK5 | 2.89E-26 | -1.28812 |
| KALRN | 4.24E-26 | -1.21777 |
| PHLPP2 | 4.62E-26 | -1.452 |
| SLC38A4 | 6.2E-26 | -4.26881 |
| TEX11 | 6.76E-26 | -2.47292 |
| ACSF2 | 7.62E-26 | -1.86135 |
| SUGCT | 7.79E-26 | -2.01658 |
| BMP3 | 9.99E-26 | -1.44786 |
| SLC52A3 | 1.09E-25 | -1.21075 |
| MOCS1 | 1.12E-25 | -1.80799 |
| LOC339166 | 1.14E-25 | -2.1531 |
| TECPR2 | 1.67E-25 | -1.4302 |
| DDAH2 | 2.25E-25 | -1.02941 |
| RHOU | 2.53E-25 | -1.43626 |
| AQP8 | 3.11E-25 | -4.83037 |
| FXYD3 | 3.11E-25 | -1.03028 |
| PAQR5 | 3.47E-25 | -1.35748 |
| HSPB3 | 3.85E-25 | -2.94775 |
| BSG | 4.27E-25 | -1.1008 |
| DEPDC7 | 5.4E-25 | -1.54015 |
| RETSAT | 5.65E-25 | -1.33548 |
| SERPINA6 | 6.16E-25 | -1.94199 |
| NAT8B | 7.46E-25 | -1.6652 |
| KCNV1 | 7.86E-25 | -1.31539 |
| RBKS | 7.95E-25 | -1.08063 |
| MOBP | 1.05E-24 | -2.05613 |
| IQGAP2 | 1.1E-24 | -1.01118 |
| ACADS | 1.35E-24 | -1.38067 |
| SERF1A | 1.47E-24 | -1.04397 |
| ANK3 | 1.47E-24 | -1.31128 |
| SLC17A8 | 1.55E-24 | -2.15629 |
| SLC1A7 | 2.63E-24 | -1.26411 |
| CMBL | 2.67E-24 | -1.23425 |
| SLC22A5 | 3.18E-24 | -1.58514 |
| HSD17B2 | 3.21E-24 | -1.57329 |
| AMACR | 3.72E-24 | -1.51596 |
| FAM151A | 3.95E-24 | -1.20086 |
| ABCB1 | 4.59E-24 | -1.37922 |
| FAM195A | 5.04E-24 | -1.1214 |
| LANCL3 | 5.04E-24 | -1.58432 |
| NAALADL1 | 5.42E-24 | -1.42684 |
| SLC36A1 | 5.57E-24 | -1.03198 |
| CYP2B6 | 6.7E-24 | -1.56288 |
| ME3 | 6.75E-24 | -1.06059 |
| ASB13 | 6.76E-24 | -1.13016 |
| ALDH18A1 | 7E-24 | -1.20178 |
| CDKN2B-AS1 | 8.6E-24 | -2.60709 |
| RUNDC3B | 9.6E-24 | -1.64946 |
| PPARG | 1.1E-23 | -1.75196 |
| G6PC | 1.14E-23 | -2.80136 |
| TGFA | 1.33E-23 | -1.19454 |
| VIL1 | 1.4E-23 | -1.1807 |
| CA1 | 1.41E-23 | -1.58387 |
| ACOT4 | 1.43E-23 | -1.27236 |
| CYP4F12 | 1.48E-23 | -1.32691 |
| DNAJB7 | 1.66E-23 | -1.77385 |
| RMDN2 | 1.67E-23 | -1.43378 |
| PLA2G12B | 1.8E-23 | -1.58904 |
| ENPP1 | 1.8E-23 | -1.28586 |
| SLC45A2 | 2.14E-23 | -1.27188 |
| SEMA5A | 2.46E-23 | -1.43747 |
| NLN | 2.85E-23 | -1.03046 |
| CNTFR | 2.93E-23 | -1.64964 |
| BTBD3 | 4.05E-23 | -1.18794 |
| NCKAP5 | 4.08E-23 | -1.22562 |
| FAM47E-STBD1 | 4.27E-23 | -1.43678 |
| ABAT | 4.84E-23 | -1.15916 |
| SLC3A1 | 5.07E-23 | -1.64194 |
| UGT1A1 | 5.27E-23 | -1.61285 |
| MYL5 | 5.38E-23 | -1.22701 |
| MYO1D | 5.38E-23 | -1.16969 |
| LGI4 | 5.5E-23 | -1.40983 |
| RPS6KA6 | 6.41E-23 | -1.2836 |
| RAPGEFL1 | 7.01E-23 | -1.24969 |
| NEURL1B | 7.34E-23 | -1.41562 |
| CES2 | 8E-23 | -1.28202 |
| SPHK2 | 1.04E-22 | -1.11791 |
| SPINT1 | 1.25E-22 | -1.20093 |
| DHRS11 | 1.26E-22 | -1.93443 |
| GCOM1 | 1.39E-22 | -1.21061 |
| SLC51A | 1.39E-22 | -4.01462 |
| NR1I2 | 1.58E-22 | -1.30979 |
| PEX11A | 1.59E-22 | -1.20173 |
| CDK20 | 1.83E-22 | -1.22101 |
| TMEM38B | 1.87E-22 | -1.04699 |
| AP3S2 | 2.03E-22 | -1.16315 |
| PXMP2 | 2.03E-22 | -1.44203 |
| CPT2 | 2.24E-22 | -1.05062 |
| PDE6A | 2.37E-22 | -2.86663 |
| GOLT1A | 2.43E-22 | -1.81477 |
| RCN3 | 2.58E-22 | -1.40741 |
| RAB6B | 2.7E-22 | -1.86534 |
| UGT2B10 | 2.7E-22 | -1.09646 |
| FRMD1 | 2.72E-22 | -1.5299 |
| CYP2B6 | 2.78E-22 | -1.38735 |
| CPT1A | 2.84E-22 | -1.16256 |
| CHP1 | 2.88E-22 | -1.16231 |
| AUH | 3.11E-22 | -1.03577 |
| ENTPD5 | 3.19E-22 | -1.26209 |
| TUBAL3 | 3.2E-22 | -2.00194 |
| B4GALNT2 | 4.79E-22 | -2.02719 |
| SFXN1 | 5.29E-22 | -1.0194 |
| SLC19A3 | 5.32E-22 | -2.00454 |
| CDKN2AIPNL | 5.32E-22 | -1.14518 |
| CDKN2B | 5.62E-22 | -1.54899 |
| AMACR | 6.25E-22 | -1.53817 |
| CRYBB2 | 6.26E-22 | -1.02674 |
| BLOC1S1-RDH5 | 6.38E-22 | -1.04573 |
| FGFR2 | 7.19E-22 | -1.63159 |
| CNNM4 | 7.34E-22 | -1.07063 |
| SLC26A2 | 7.37E-22 | -3.48196 |
| WDR78 | 8.13E-22 | -1.26232 |
| TRHDE | 8.32E-22 | -2.14525 |
| LTK | 8.53E-22 | -1.04933 |
| MROH7 | 9.73E-22 | -1.54646 |
| PLCE1 | 1.05E-21 | -1.17488 |
| TMEM253 | 1.05E-21 | -1.17172 |
| INPP5J | 1.13E-21 | -1.12501 |
| CTSV | 1.15E-21 | -1.38651 |
| SLC16A9 | 1.38E-21 | -2.24349 |
| GLB1L2 | 1.63E-21 | -1.4615 |
| PLEKHG6 | 1.85E-21 | -1.53276 |
| MARVELD3 | 1.94E-21 | -1.354 |
| TMEM171 | 1.95E-21 | -1.31037 |
| EDA | 1.95E-21 | -1.56211 |
| LUZP2 | 2.16E-21 | -2.50717 |
| PDZD3 | 2.43E-21 | -1.85108 |
| MPST | 2.54E-21 | -1.02577 |
| ANPEP | 2.75E-21 | -2.82029 |
| CLCN2 | 3.08E-21 | -1.18376 |
| ACKR2 | 3.5E-21 | -1.38399 |
| SLC6A4 | 3.63E-21 | -1.0787 |
| CBS | 3.7E-21 | -1.63438 |
| SLC39A2 | 3.96E-21 | -2.2115 |
| EHHADH | 4.13E-21 | -1.1485 |
| PLIN1 | 4.4E-21 | -2.01724 |
| MPP7 | 4.48E-21 | -1.03681 |
| SHOX | 4.7E-21 | -1.05485 |
| RAVER2 | 4.8E-21 | -1.019 |
| SRI | 5.49E-21 | -1.08879 |
| SLC17A4 | 5.71E-21 | -1.94418 |
| CELF5 | 5.73E-21 | -1.4524 |
| TNFRSF1A | 6.15E-21 | -1.05238 |
| PPP2R3A | 6.8E-21 | -1.08383 |
| C1orf115 | 1.07E-20 | -1.25914 |
| SLC16A1 | 1.22E-20 | -2.2503 |
| C7orf31 | 1.49E-20 | -1.21953 |
| TMEM56 | 1.52E-20 | -1.32416 |
| CYP27A1 | 1.62E-20 | -1.21659 |
| TAT | 1.75E-20 | -1.61976 |
| MYZAP | 1.82E-20 | -1.15007 |
| ITPKA | 1.91E-20 | -1.32063 |
| SLC10A2 | 1.96E-20 | -1.72575 |
| MAPRE3 | 2.09E-20 | -1.15388 |
| GPR39 | 2.09E-20 | -1.10732 |
| ETHE1 | 2.13E-20 | -1.05866 |
| PCK1 | 2.27E-20 | -2.90753 |
| ALPI | 2.4E-20 | -1.48281 |
| BDH1 | 2.55E-20 | -1.07585 |
| TSEN2 | 2.91E-20 | -1.07035 |
| CHP2 | 2.92E-20 | -2.53878 |
| ELAVL2 | 2.97E-20 | -1.75812 |
| TSPAN7 | 3E-20 | -1.29262 |
| MTMR11 | 3.14E-20 | -1.08837 |
| CDK3 | 3.19E-20 | -1.06518 |
| COBL | 3.37E-20 | -1.07467 |
| GLYCTK | 3.5E-20 | -1.0366 |
| LIPC | 3.7E-20 | -1.99251 |
| FSIP1 | 4.43E-20 | -1.71274 |
| CNTN4 | 4.74E-20 | -1.21348 |
| CLIP2 | 6.24E-20 | -1.09263 |
| HNF4A | 6.71E-20 | -1.24784 |
| TPRN | 7.34E-20 | -1.087 |
| PLCD1 | 7.49E-20 | -1.49181 |
| DDC | 7.79E-20 | -1.14926 |
| CA12 | 8.59E-20 | -1.66742 |
| PHYH | 8.74E-20 | -1.12268 |
| EPB41L5 | 9.72E-20 | -1.06761 |
| CELA3A | 9.91E-20 | -2.86887 |
| VLDLR | 1.04E-19 | -1.89614 |
| OAF | 1.11E-19 | -1.33984 |
| TNMD | 1.18E-19 | -1.43387 |
| VIPR1 | 1.18E-19 | -1.19845 |
| HMGCS2 | 1.22E-19 | -2.60376 |
| PDK2 | 1.22E-19 | -1.52945 |
| TLN2 | 1.23E-19 | -1.02391 |
| IGSF9 | 1.39E-19 | -1.88815 |
| YBX2 | 1.4E-19 | -1.31873 |
| KLB | 1.46E-19 | -1.50032 |
| BCL2L10 | 1.96E-19 | -1.44087 |
| PGAP3 | 2.27E-19 | -1.06431 |
| MIER3 | 2.49E-19 | -1.5505 |
| MXI1 | 2.52E-19 | -1.31163 |
| PTPRF | 2.94E-19 | -1.02948 |
| PEX26 | 3.08E-19 | -1.07697 |
| LRRC75A | 3.27E-19 | -1.11372 |
| SLC51B | 3.35E-19 | -1.67316 |
| ESPN | 4.36E-19 | -1.50861 |
| CYP4F2 | 5.02E-19 | -1.22135 |
| APOH | 5.13E-19 | -1.13598 |
| XK | 5.59E-19 | -1.08478 |
| SYN3 | 5.73E-19 | -1.03493 |
| GNA11 | 6.28E-19 | -1.16812 |
| DPP10 | 6.54E-19 | -1.84534 |
| CYP2B7P | 6.95E-19 | -3.00799 |
| DEFB1 | 7E-19 | -2.33049 |
| EDN1 | 8.01E-19 | -1.16839 |
| PPFIA3 | 8.42E-19 | -1.21011 |
| NPY | 8.52E-19 | -1.68183 |
| FMO4 | 9.12E-19 | -1.2196 |
| MSI1 | 9.29E-19 | -1.13563 |
| CRYL1 | 1.07E-18 | -1.03795 |
| AGFG2 | 1.12E-18 | -1.61758 |
| ADIRF | 1.44E-18 | -1.44603 |
| GBA3 | 1.48E-18 | -2.79829 |
| STEAP3 | 1.53E-18 | -1.00693 |
| EDN2 | 1.84E-18 | -1.32082 |
| CNTNAP2 | 1.94E-18 | -1.19732 |
| MAST2 | 2.3E-18 | -1.13676 |
| CLU | 2.68E-18 | -1.70439 |
| NDRG1 | 2.92E-18 | -1.39279 |
| UGDH | 2.93E-18 | -1.1114 |
| PRAP1 | 3E-18 | -2.65803 |
| LOC729966 | 3.03E-18 | -1.6027 |
| SLC25A23 | 3.22E-18 | -1.15022 |
| GUCA2B | 4.16E-18 | -2.65734 |
| ACOX2 | 5.44E-18 | -1.17961 |
| DAO | 5.96E-18 | -1.45361 |
| HSD3B2 | 6.56E-18 | -2.824 |
| KCNG1 | 6.67E-18 | -1.05767 |
| CLYBL | 6.83E-18 | -1.09555 |
| APPL2 | 6.95E-18 | -1.02242 |
| ERBB2 | 9.2E-18 | -1.01775 |
| HSD17B3 | 9.98E-18 | -1.30195 |
| APOBEC3A | 1.09E-17 | -1.63416 |
| ADORA2B | 1.09E-17 | -1.00342 |
| ZBTB7C | 1.17E-17 | -1.09803 |
| MESP1 | 1.19E-17 | -1.10169 |
| PKIB | 1.19E-17 | -1.51832 |
| GDPD2 | 1.35E-17 | -1.90702 |
| GXYLT2 | 1.42E-17 | -1.52187 |
| CHN2 | 1.49E-17 | -1.48743 |
| TRIM36 | 1.85E-17 | -1.24662 |
| ETNK1 | 1.87E-17 | -1.55408 |
| C2orf54 | 2.16E-17 | -1.25285 |
| MAGI1 | 2.18E-17 | -1.0337 |
| EPB41L4B | 2.27E-17 | -1.31214 |
| CHRNA1 | 2.42E-17 | -1.81582 |
| CNNM2 | 2.52E-17 | -1.20546 |
| SLC30A10 | 2.56E-17 | -1.89009 |
| HTR3C | 2.59E-17 | -1.14764 |
| P2RX4 | 2.98E-17 | -1.00688 |
| SLC46A1 | 3.44E-17 | -1.02904 |
| TMEM37 | 4.16E-17 | -1.4999 |
| GJB1 | 4.29E-17 | -1.11192 |
| HPGD | 4.95E-17 | -1.13885 |
| MT3 | 4.95E-17 | -1.13295 |
| NHEJ1 | 5.11E-17 | -1.09658 |
| SCUBE2 | 5.5E-17 | -1.56109 |
| EDN3 | 5.94E-17 | -1.37733 |
| AIFM3 | 6.04E-17 | -1.63041 |
| TINAG | 6.19E-17 | -1.52418 |
| ACSS2 | 6.81E-17 | -1.06741 |
| TM6SF2 | 6.83E-17 | -1.4011 |
| DUSP21 | 7.07E-17 | -1.04774 |
| ARHGAP44 | 7.2E-17 | -1.19664 |
| SHD | 7.49E-17 | -1.18305 |
| BTNL3 | 9.42E-17 | -1.18672 |
| SLC39A5 | 9.93E-17 | -1.31769 |
| STAP2 | 1.09E-16 | -1.05066 |
| EFNA1 | 1.19E-16 | -1.27787 |
| SLC22A18AS | 1.27E-16 | -1.16857 |
| OTC | 1.28E-16 | -1.97633 |
| FREM2 | 1.29E-16 | -1.02722 |
| CPA2 | 1.39E-16 | -1.20384 |
| SVOPL | 1.46E-16 | -1.74433 |
| SLC4A4 | 1.51E-16 | -1.01523 |
| KIAA2022 | 1.61E-16 | -1.31999 |
| AVIL | 1.74E-16 | -1.02376 |
| LRRC19 | 1.8E-16 | -1.31388 |
| PLIN2 | 1.91E-16 | -1.28014 |
| FCGRT | 1.92E-16 | -1.07478 |
| DQX1 | 2.01E-16 | -1.02359 |
| NRG4 | 2.13E-16 | -1.4743 |
| CNGA1 | 2.25E-16 | -1.79217 |
| MIPOL1 | 2.61E-16 | -1.5549 |
| C11orf52 | 2.69E-16 | -1.1442 |
| AQP12A | 2.74E-16 | -1.36746 |
| GDA | 2.98E-16 | -1.13077 |
| ABLIM2 | 3.09E-16 | -1.03855 |
| C9orf24 | 3.11E-16 | -1.04595 |
| ADPRHL1 | 3.86E-16 | -1.07644 |
| FAM47E | 3.92E-16 | -1.19058 |
| SGK223 | 4.17E-16 | -1.13256 |
| AKR1B10 | 5.35E-16 | -1.27161 |
| PLA2R1 | 5.53E-16 | -1.01176 |
| ABHD5 | 5.6E-16 | -1.03023 |
| SEMA6A | 6.37E-16 | -1.0329 |
| TMCC3 | 6.56E-16 | -1.04116 |
| SPIRE2 | 7.23E-16 | -1.07861 |
| GRPR | 9.96E-16 | -1.12471 |
| HOXA6 | 1.06E-15 | -1.11072 |
| RAB17 | 1.08E-15 | -1.01784 |
| RPL10L | 1.12E-15 | -1.10488 |
| NPY1R | 1.36E-15 | -1.4671 |
| TP53TG1 | 1.38E-15 | -1.08893 |
| AKR1B15 | 1.44E-15 | -1.14527 |
| MEP1A | 1.49E-15 | -1.40212 |
| MOGAT3 | 1.63E-15 | -1.0206 |
| ERBB3 | 1.64E-15 | -1.03318 |
| CCDC183 | 1.97E-15 | -1.6298 |
| RIMKLA | 2E-15 | -1.34314 |
| KRT12 | 2.05E-15 | -2.12591 |
| PPARGC1A | 2.16E-15 | -1.34987 |
| MT1F | 2.18E-15 | -1.20239 |
| AKR1B10 | 2.31E-15 | -1.05137 |
| KCNG3 | 2.41E-15 | -1.21367 |
| RBP2 | 2.59E-15 | -1.34575 |
| CNKSR3 | 2.72E-15 | -1.0429 |
| ADH6 | 2.97E-15 | -1.21202 |
| MOGAT2 | 3.05E-15 | -1.05032 |
| ABCC13 | 3.51E-15 | -1.67479 |
| SLC4A10 | 3.52E-15 | -1.62841 |
| SHISA9 | 4.05E-15 | -1.27036 |
| PBLD | 4.4E-15 | -1.59071 |
| KANK4 | 4.43E-15 | -1.30062 |
| GNG4 | 4.49E-15 | -1.69257 |
| SCIN | 5.08E-15 | -1.22535 |
| UGT2A3 | 5.53E-15 | -1.82613 |
| MYO1A | 5.75E-15 | -1.06046 |
| VSTM2A | 6.2E-15 | -1.92939 |
| NWD2 | 6.56E-15 | -1.51419 |
| THBS1 | 7.02E-15 | -1.11744 |
| PPP1R14C | 7.27E-15 | -1.30371 |
| TRPM4 | 7.34E-15 | -1.06319 |
| BAIAP2L2 | 7.6E-15 | -1.0065 |
| PTPRR | 1.06E-14 | -1.37422 |
| MAOA | 1.06E-14 | -1.35875 |
| CYP2S1 | 1.09E-14 | -1.01744 |
| ISX | 1.12E-14 | -1.28726 |
| LRRC31 | 1.37E-14 | -1.21805 |
| ATOH7 | 1.38E-14 | -1.09433 |
| GRAMD3 | 1.54E-14 | -1.00565 |
| CGN | 1.61E-14 | -1.12149 |
| CROT | 1.61E-14 | -1.00902 |
| ZNF385B | 1.84E-14 | -1.07408 |
| PKDCC | 1.9E-14 | -1.17559 |
| HAVCR1 | 1.91E-14 | -1.16501 |
| USP2 | 2.01E-14 | -1.35383 |
| FAM189A1 | 2.06E-14 | -1.73364 |
| PIGZ | 2.12E-14 | -1.55087 |
| CDHR5 | 2.16E-14 | -1.2871 |
| KCNK10 | 2.36E-14 | -1.01862 |
| NETO2 | 2.49E-14 | -1.64654 |
| SSTR1 | 2.95E-14 | -1.23768 |
| ABCA8 | 3.11E-14 | -1.69851 |
| CDH13 | 3.11E-14 | -1.0652 |
| SLC13A2 | 3.23E-14 | -1.29 |
| GRAMD1C | 3.32E-14 | -1.14619 |
| GPR37L1 | 3.51E-14 | -1.42232 |
| RNF152 | 3.73E-14 | -1.25091 |
| SELENBP1 | 4.21E-14 | -1.20596 |
| CYP4F2 | 4.4E-14 | -1.13974 |
| TLE2 | 4.4E-14 | -1.002 |
| ADAMTSL1 | 4.54E-14 | -1.05821 |
| KLK3 | 4.86E-14 | -1.17126 |
| P2RY1 | 5.64E-14 | -1.42618 |
| SLC5A11 | 6.26E-14 | -1.12492 |
| CWH43 | 6.95E-14 | -2.1991 |
| EXPH5 | 9.12E-14 | -1.34223 |
| ARHGEF37 | 9.44E-14 | -1.02191 |
| HOXA5 | 1.1E-13 | -1.13101 |
| RNF157 | 1.16E-13 | -1.24759 |
| MB | 1.28E-13 | -1.2734 |
| APOBEC3B | 1.33E-13 | -1.72107 |
| THRB | 1.35E-13 | -1.05073 |
| MGLL | 1.48E-13 | -1.03213 |
| SOWAHA | 1.52E-13 | -1.14217 |
| TMIGD1 | 1.54E-13 | -2.3321 |
| SSTR2 | 1.68E-13 | -1.28531 |
| TMEM252 | 1.98E-13 | -1.01119 |
| ZG16 | 2.11E-13 | -1.64952 |
| CEACAM8 | 2.14E-13 | -1.16793 |
| IHH | 2.54E-13 | -1.08567 |
| GUCA2A | 2.85E-13 | -2.01045 |
| CHAD | 3.16E-13 | -1.55544 |
| ACVR1C | 3.33E-13 | -1.54131 |
| SETD9 | 3.51E-13 | -1.12206 |
| TCEA3 | 4.52E-13 | -1.09417 |
| NR1H4 | 5.28E-13 | -2.41973 |
| MCOLN2 | 5.3E-13 | -1.03585 |
| MMP28 | 5.31E-13 | -1.07103 |
| PITX2 | 5.94E-13 | -3.73061 |
| MT1H | 6.27E-13 | -1.64169 |
| FAM135B | 6.6E-13 | -1.0889 |
| SATB2 | 7.5E-13 | -1.09534 |
| AGMO | 7.77E-13 | -1.49538 |
| CYP4F3 | 7.96E-13 | -1.15032 |
| C2orf88 | 1.24E-12 | -1.18232 |
| C6orf222 | 1.44E-12 | -1.04004 |
| CELP | 1.5E-12 | -1.40298 |
| TMEM236 | 1.54E-12 | -1.37673 |
| EMP1 | 2.42E-12 | -1.02899 |
| TRPM6 | 2.78E-12 | -2.0677 |
| MT1G | 2.87E-12 | -1.02901 |
| KCTD4 | 3.32E-12 | -1.26334 |
| LDHD | 3.79E-12 | -1.4368 |
| BEST4 | 4.72E-12 | -2.01463 |
| TM4SF5 | 4.87E-12 | -1.07777 |
| ASXL3 | 4.89E-12 | -1.08343 |
| NAGS | 5.17E-12 | -1.14622 |
| ASPA | 5.41E-12 | -1.14324 |
| CDHR1 | 5.88E-12 | -1.6297 |
| LAMA1 | 7.46E-12 | -1.67112 |
| AKR7L | 8.28E-12 | -1.014 |
| CYP2C9 | 9.17E-12 | -1.31501 |
| SLC22A4 | 1.16E-11 | -1.25925 |
| LRIT2 | 1.29E-11 | -1.38391 |
| MUC20 | 1.33E-11 | -1.25473 |
| A1CF | 1.37E-11 | -1.0554 |
| NXPE2 | 1.62E-11 | -1.24405 |
| CAPN13 | 1.86E-11 | -1.86237 |
| HAS3 | 2.59E-11 | -1.71254 |
| PTGDR | 2.76E-11 | -1.06801 |
| FMO5 | 3.41E-11 | -1.03043 |
| LINC01559 | 3.98E-11 | -1.68871 |
| HRCT1 | 4.43E-11 | -1.31353 |
| XPNPEP2 | 5.77E-11 | -1.69312 |
| TMEM72 | 5.83E-11 | -1.83722 |
| SLCO4C1 | 7.08E-11 | -1.02424 |
| LOC102724562 | 8.7E-11 | -1.6055 |
| CA7 | 8.96E-11 | -1.68079 |
| BEST2 | 9.21E-11 | -1.7964 |
| SLC22A3 | 9.27E-11 | -1.18082 |
| MT1JP | 1.05E-10 | -1.11759 |
| OASL | 1.27E-10 | -1.07656 |
| GSTA1 | 1.33E-10 | -1.44673 |
| PNLIPRP2 | 1.96E-10 | -1.90442 |
| DISP2 | 1.98E-10 | -1.11267 |
| LHFPL3 | 2.18E-10 | -1.38638 |
| CYP3A4 | 2.58E-10 | -1.31009 |
| CKB | 3.74E-10 | -1.57661 |
| LYPD8 | 5.59E-10 | -1.23826 |
| EYA2 | 1.03E-09 | -1.28775 |
| MT1JP | 1.19E-09 | -1.83148 |
| CNTN3 | 1.2E-09 | -1.16508 |
| LOC105376948 | 1.2E-09 | -1.15636 |
| HTR3E | 1.35E-09 | -1.23719 |
| BRINP3 | 1.49E-09 | -1.36423 |
| MT1M | 1.84E-09 | -1.52475 |
| FAM150B | 1.9E-09 | -1.0799 |
| ADH1C | 1.98E-09 | -1.03784 |
| DMRTA1 | 2.01E-09 | -1.26398 |
| GULP1 | 2.22E-09 | -1.06999 |
| ATP6V0D2 | 2.24E-09 | -1.06461 |
| SLC1A1 | 3.35E-09 | -1.16144 |
| KCNE2 | 4.02E-09 | -1.18149 |
| NXPE4 | 4.16E-09 | -1.10769 |
| ENPEP | 7.94E-09 | -1.00726 |
| CHDH | 7.94E-09 | -1.00461 |
| HEPACAM2 | 1.25E-08 | -1.08191 |
| PLP1 | 1.84E-08 | -1.12385 |
| CEACAM7 | 3.25E-08 | -1.28335 |
| GABRA2 | 3.41E-08 | -1.16833 |
| FAM183A | 4.37E-08 | -1.05002 |
| SLC6A19 | 6.04E-08 | -1.29566 |
| KLK15 | 1.06E-07 | -1.02326 |
| CD177 | 1.35E-07 | -1.97629 |
| TRIM9 | 1.59E-07 | -1.04206 |
| TMED6 | 1.74E-07 | -1.11785 |
| FABP2 | 2.01E-07 | -1.04943 |
| SH2D6 | 2.92E-07 | -1.17708 |
| CEACAM1 | 4.85E-07 | -1.01401 |
| ERVK3-2 | 5.18E-07 | -1.14172 |
| DEPDC5 | 7E-07 | -1.00029 |
| TRPV3 | 8.38E-07 | -1.05269 |
| SLC37A2 | 8.99E-07 | -1.08959 |
| POPDC3 | 1.06E-06 | -1.033 |
| CLDN8 | 1.95E-06 | -2.59125 |
| SCNN1B | 6.72E-06 | -1.14584 |
| NTRK2 | 1.65E-05 | -1.06636 |
| KNG1 | 1.97E-05 | -1.09003 |
| SCN9A | 3.45E-05 | -1.06239 |
| PCDH20 | 0.000512 | -1.02925 |

**Table S3.** Topological analysis in PPI network

| Target | Degree | Betweenness | Closeness |
| --- | --- | --- | --- |
| IL6 | 45 | 684.4984 | 0.29411766 |
| TNF | 45 | 779.93317 | 0.29182878 |
| IL1B | 40 | 400.18173 | 0.28625953 |
| PTGS2 | 33 | 523.231 | 0.2788104 |
| MMP9 | 33 | 260.1599 | 0.2747253 |
| CCL2 | 32 | 134.04642 | 0.2757353 |
| ESR1 | 31 | 740.9644 | 0.27272728 |
| VCAM1 | 27 | 92.65536 | 0.26408452 |
| KDR | 25 | 174.57202 | 0.26595744 |
| IFNG | 24 | 65.20235 | 0.2669039 |
| PPARG | 23 | 370.8105 | 0.2669039 |
| SERPINE1 | 23 | 45.263935 | 0.26223776 |
| MMP1 | 20 | 26.919462 | 0.25862068 |
| SELE | 20 | 25.283207 | 0.25773194 |
| MMP3 | 20 | 29.698782 | 0.25862068 |
| COL1A1 | 19 | 126.071144 | 0.25862068 |
| STAT1 | 19 | 38.07298 | 0.26132405 |
| PDGFRB | 19 | 129.62054 | 0.25684932 |
| TNFRSF1A | 18 | 10.62183 | 0.2542373 |
| PLAU | 17 | 11.274889 | 0.25510204 |
| MMP7 | 16 | 19.996086 | 0.25510204 |
| CYP3A4 | 15 | 264.08136 | 0.2533784 |
| CXCR2 | 15 | 90.85626 | 0.25083613 |
| NOS2 | 14 | 287.0907 | 0.25167784 |
| MMP10 | 14 | 10.719313 | 0.24834438 |
| CTSK | 13 | 65.07744 | 0.24834438 |
| CASP1 | 12 | 0.5 | 0.24834438 |
| CXCR1 | 12 | 61.866943 | 0.24429968 |
| LCK | 12 | 18.653141 | 0.24590164 |
| ABCB1 | 11 | 31.757565 | 0.25083613 |
| CYP2C9 | 11 | 76.29152 | 0.24350649 |
| NR1H4 | 11 | 85.808556 | 0.24671052 |
| NOX4 | 11 | 0.14874142 | 0.24752475 |
| MMP12 | 11 | 1.2518148 | 0.24509804 |
| JAK3 | 11 | 13.75364 | 0.24115756 |
| FYN | 11 | 14.664024 | 0.24271844 |
| SLC6A4 | 9 | 159.75836 | 0.24752475 |
| THBD | 9 | 0.16666667 | 0.2388535 |
| IDO1 | 9 | 62.873436 | 0.24350649 |
| CD38 | 9 | 2.7982905 | 0.2388535 |
| ABCG2 | 8 | 31.070547 | 0.23219815 |
| CYP2B6 | 8 | 15.696218 | 0.23291926 |
| NTRK2 | 8 | 57.354816 | 0.24671052 |
| ADORA2A | 7 | 148.29683 | 0.23961662 |
| FPR2 | 7 | 0.7459014 | 0.23961662 |
| PLA2G2A | 7 | 13.052753 | 0.24350649 |
| PIK3CD | 7 | 5.171022 | 0.22522523 |
| MME | 6 | 2.191111 | 0.24271844 |
| PRSS1 | 6 | 0.125 | 0.22058824 |
| HSD11B1 | 5 | 145.23215 | 0.24350649 |
| ADORA2B | 4 | 0 | 0.23364486 |
| MGLL | 4 | 146.0619 | 0.23076923 |
| GSTA1 | 4 | 0 | 0.21008404 |
| HSD17B2 | 4 | 3.5333333 | 0.2238806 |
| STS | 4 | 3.5333333 | 0.2238806 |
| MAOA | 4 | 22.94386 | 0.2173913 |
| FGFR1 | 4 | 3.4007015 | 0.2265861 |
| CES2 | 3 | 0 | 0.20775624 |
| ALPL | 3 | 0.7532467 | 0.22058824 |
| DNM1 | 3 | 14.625389 | 0.20547946 |
| GABRA2 | 3 | 9.015894 | 0.20380434 |
| HSD17B3 | 3 | 0 | 0.2173913 |
| DAO | 2 | 144 | 0.20380434 |
| FAP | 2 | 0 | 0.21367522 |
| CTSV | 2 | 0 | 0.2186589 |
| ABAT | 1 | 0 | 0.17045455 |
| PDE10A | 1 | 0 | 0.19480519 |
| AKR1B10 | 1 | 0 | 0.18891688 |
| CA1 | 1 | 0 | 0.013333334 |
| CA7 | 1 | 0 | 0.013333334 |
| CA12 | 1 | 0 | 0.21613833 |
| EDNRA | 1 | 0 | 0.22796352 |
| FADS1 | 1 | 0 | 0.21246459 |
| SERPINA6 | 1 | 0 | 0.19736843 |
| PDE4B | 1 | 0 | 0.22796352 |
| PTGFR | 1 | 0 | 0.21994135 |

|  |
| --- |

|  |
| --- |
